# Chronic in vivo imaging defines age-dependent alterations of neurogenesis in the mouse hippocampus

**DOI:** 10.1038/s43587-023-00370-9

**Published:** 2023-02-20

**Authors:** Yicheng Wu, Sara Bottes, Roberto Fisch, Cinzia Zehnder, John Darby Cole, Gregor-Alexander Pilz, Fritjof Helmchen, Benjamin D. Simons, Sebastian Jessberger

**Affiliations:** 1 Laboratory of Neural Plasticity, Faculties of Medicine and Science, Brain Research Institute, University of Zurich, Zurich, Switzerland; 2 Laboratory of Neural Circuit Dynamics, Faculties of Medicine and Science, Brain Research Institute, University of Zurich, Zurich, Switzerland; 3 Wellcome Trust-Medical Research Council Stem Cell Institute, University of Cambridge, Cambridge, UK; 4 Wellcome Trust/Cancer Research UK Gurdon Institute, University of Cambridge, Cambridge, UK; 5 Department of Applied Mathematics and Theoretical Physics, Centre for Mathematical Sciences, University of Cambridge, Cambridge, UK; 6 Present Address: BioMedical Center, Department of Cell Biology and Anatomy, Ludwig Maximilians University, Planegg-Martinsried, Germany

**Keywords:** Neural stem cells, Neural stem cells

## Abstract

Neural stem cells (NSCs) generate new neurons throughout life in the mammalian hippocampus^1^. Advancing age leads to a decline in neurogenesis, which is associated with impaired cognition^2,3^. The cellular mechanisms causing reduced neurogenesis with advancing age remain largely unknown. We genetically labeled NSCs through conditional recombination driven by the regulatory elements of the stem-cell-expressed gene GLI family zinc finger 1 (Gli1) and used chronic intravital imaging to follow individual NSCs and their daughter cells over months within their hippocampal niche^4,5^. We show that aging affects multiple steps, from cell cycle entry of quiescent NSCs to determination of the number of surviving cells, ultimately causing reduced clonal output of individual NSCs. Thus, we here define the developmental stages that may be targeted to enhance neurogenesis with the aim of maintaining hippocampal plasticity with advancing age.

## Abstract (web summary)

Using chronic in vivo imaging of the mouse hippocampus over months, this study reveals aging-associated alterations of neural stem cells and their neuronal progeny that lead to reduced clonal output of individual neural stem cells with advancing age.

## Main

Throughout life, NSCs in the hippocampal dentate gyrus (DG) give rise to new neurons that are involved in DG-dependent brain function^1–3^. The number of newborn neurons is dynamically regulated and has been associated with the etiology of numerous diseases affecting the hippocampus, including major depression and cognitive aging^3,6^. Indeed, advancing age is associated with a dramatic decrease in the rate of hippocampal neurogenesis, which drops by around 80% from 2 to 8 months of age in the rodent brain before it plateaus and continues throughout the entire lifespan, albeit at low levels^7–10^. Levels of neurogenesis and performance in hippocampus-dependent behavior are correlated in rodents, and recent evidence suggests that an age-dependent and neurodegeneration-associated decrease in neurogenesis may also occur in the human hippocampus^1,11–14^. The NSC pool is reduced in middle-aged mice (around 12 months of age) when neurogenesis has sharply dropped^9,15,16^. Reduced NSC numbers and subsequently reduced levels of neurogenesis may be due to NSC exhaustion, altered NSC fate, enhanced quiescence or altered cell death caused by cell-intrinsic stem cell aging and niche-dependent mechanisms^4,7,17–23^. Owing to heterogeneity of clonal behavior and poor temporal resolution, the recovery of lineage information from static pulse-chase lineage tracing assays such as Cre-mediated lineage tracing is, by definition, ambiguous and uncertain. However, defining the cellular principles that mediate the age-dependent drop in neurogenesis in the DG is a prerequisite for targeted enhancement of neurogenesis in the aging brain^24–27^. Thus, we used chronic intravital imaging to record individual NSC lineages and analyzed their fate behavior, clonal output and neuronal maturation within the endogenous hippocampal niche in young (2-month-old, 2MO) and middle-aged (12MO) mice.

## Dormancy and neurogenic output of middle-aged NSCs

**Fig. 1:**
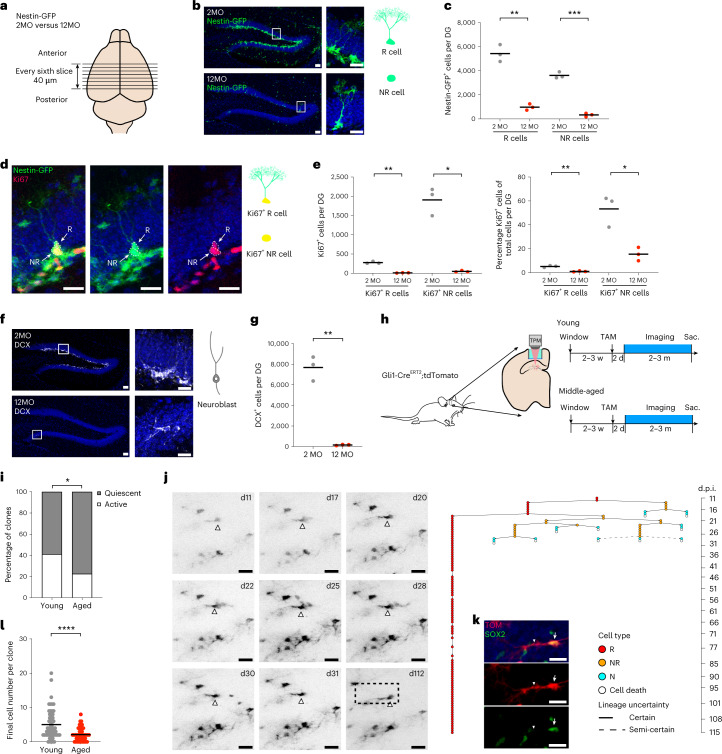
Increased dormancy and decreased neurogenic output of hippocampal NSCs at middle age. **a**, Schematic experimental illustration of age-related changes in adult mouse hippocampal neurogenesis using Nestin-GFP mouse line. **b**, Representative immunofluorescence images of Nestin-GFP-labeled NSCs (both R and NR cells). **c**, Quantification of the numbers of R and NR cells in the DG of young and middle-aged mice (R cells: 2MO 5435 ± 707 cells, 12MO 980 ± 265 cells, two-tailed unpaired *t* test with Welch’s correction, **P = 0.0039, *t* = 10.22, d.f. = 2.551; NR cells: 2MO 3613 ± 265 cells, 12MO 329 ± 147 cells, two-tailed unpaired *t* test with Welch’s correction, ***P = 0.0003, *t* = 18.79, d.f. = 3.119, for the comparison of NR cells; *n* = 3 for each age). **d**, Representative immunofluorescence images of Ki67-labeled proliferating NSCs. **e**, Left, quantification of the number of Ki67^+^ NSCs in the DG of young and middle-aged mice (Ki67^+^ R cells: 2MO 277 ± 30 cells, 12MO 10.3 ± 4.7 cells, two-tailed unpaired *t* test with Welch’s correction, **P = 0.0036, *t* = 15.04, d.f. = 2.097; Ki67^+^ NR cells: 2MO 1906 ± 362 cells, 12MO 47.7 ± 21.1 cells, two-tailed unpaired *t* test with Welch’s correction, *P = 0.0122, *t* = 8.871, d.f. = 2.014; *n* = 3 for each age). Right, percentages of Ki67^+^ NSCs in the DG of young and middle-aged mice (Ki67^+^ R cells: 2MO 5.2 ± 0.9% Ki67^+^ R cells/total R cells, 12MO 1.0 ± 0.4% Ki67^+^ R cells/total R cells, two-tailed unpaired *t* test with Welch’s correction, **P = 0.0044, *t* = 7.578, d.f. = 3.076; Ki67^+^ NR cells: 2MO 53.4 ± 13.2% Ki67^+^ NR cells/total NR cells, 12MO 15.5 ± 5.1% Ki67^+^ NR cells/total NR cells, two-tailed unpaired *t* test with Welch’s correction, *P = 0.0247, *t* = 4.565, d.f. = 2.697; *n* = 3 for each age). **f**, Representative immunofluorescence images of DCX-labeled neuronal progeny. **g**, Quantification of numbers of DCX^+^ cells in the DG of young and middle-aged mice (2MO: 7685 ± 1189 DCX^+^ cells; 12MO: 163 ± 55 DCX^+^ cells; two-tailed unpaired *t* test with Welch’s correction, **P = 0.0081, *t* = 10.95, d.f. = 2.009, *n* = 3 for each age). **h**, Schematic experimental design of chronic intravital imaging of Gli1-targeted NSCs. Sac, sacrificed. **i**, Percentages of active and quiescent Gli1-targeted NSCs in young and middle-aged mice (young: 41.2%; middle-aged: 22.6%) Two-sided Fisher’s exact test, *P* = 0.0003. **j**, Representative active clone in middle-aged mice. Left, representative images (collapsed z-stacks) of a Gli1-targeted R cell (indicated by arrowhead) and its progeny imaged over 100 days. Right, corresponding lineage tree. **k**, Post hoc immunofluorescence images of the resting R cell from **j** with cell body indicated by arrow and radial process by arrowhead. **l**, Final number of cells per active clone (young: 5.0 ± 4.3 cells, *n* = 56 clones from six mice; middle-aged: 2.1 ± 1.9 cells, *n* = 47 clones from five mice); two-tailed unpaired *t* test with Welch’s correction, ****P < 0.0001, *t* = 4.538, d.f. = 78.73. d, day; m, month; N, neuron; TPM, two-photon microscopy; w, week. All data are presented as mean ± s.e.m. Scale bars, 20 μm. NS, not significant; *P* > 0.05; *P < 0.05, **P < 0.01, ***P < 0.001, ****P < 0.0001. For detailed statistics, see Supplementary Table 1Source Data. Source Data

**Extended Data Fig. 1:**
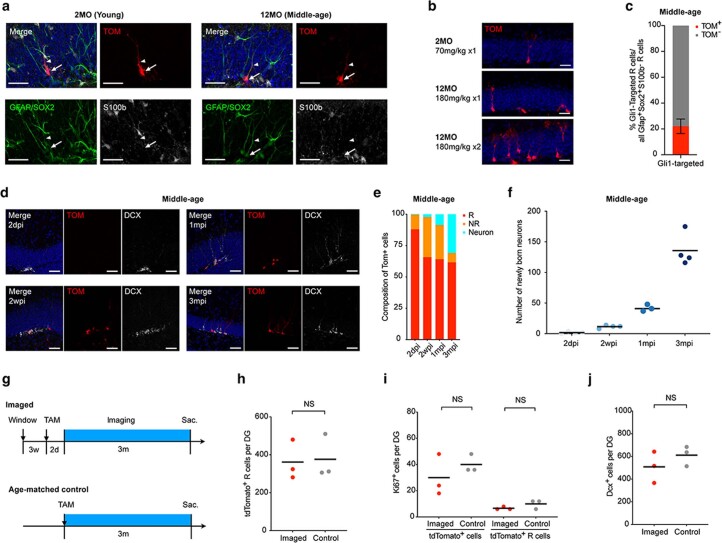
Gli1-targeted R cells comprise a substantial proportion of qNSCs in the middle-aged mice and contribute to active neurogenesis over time that is not affected by chronic hippocampal window implantation and isoflurane exposure. **a**, Representative immunofluorescence images of a Gli1-targeted R cell (GFAP^+^SOX2^+^tdTOM^+^S100b^−^) after TAM recombination in both young and middle-aged mice show conservative expression profile of feature markers and morphlogy between two ages. **b**, One single dose of TAM sparsely labels R cells in the young (70 mg/kg, upper) and middle-aged (180 mg/kg, middle) mice and the number of targeted R cells can be further increased with repeated TAM induction (180 mg/kg x2, lower). **c**, The percentage of tdTOM^+^ R cells/all R cells in the middle-aged DG after 2 TAM induction (22.1 ± 5.62%, n = 3 mice). **d**, Gli1-targeted R cells contribute to active neurogenesis over the time course. **e**, Quantification of the composition of tdTOM^+^ cells at different time points after TAM induction (2dpi: R 88.0%, NR 11.7%, Neuron 0.3%; 2wpi: R 65.8%, NR 32.0%, Neuron 2.2%; 1mpi: R 64.1%, NR 27.3%, Neuron 8.6%; 3mpi: R 61.6%, NR 7.5%, Neuron 30.9%). **f**, The number of tdTOM^+^ newly born neurons increases over the time course from 2dpi to 3mpi (2dpi, n = 4 mice; 2wpi, n = 4 mice; 1mpi, n = 3 mice; 3mpi, n = 4 mice). **g**, Control experiment studying the effects of chronic hippocampal window implantation and isoflurane exposure on hippocampal neurogenesis in mice with advanced age. **h-j**, Quantitation shows no significant difference in the total number of Gli1-targeted NSCs (tdTOM^+^ R cells, imaged: 361.7 ± 104.7 cells; control: 376.0 ± 116.1 cells; two-tailed unpaired t-test, NS, t = 0.1583, df = 3.958, n = 3 for each group) (**h**), proliferating NSCs (Ki67^+^ tdTOM^+^ cells, imaged: 24 ± 27.5 cells; control: 30 ± 26.2 cells, two-tailed unpaired t-test, NS, p = 0.8820, t = 0.1581, df = 2.439, n = 3 individuals of each group; Ki67^+^ tdTOM^+^ R cells, imaged: 4 ± 3.5 cells; control: 2 ± 3.5 cells, two-tailed unpaired t-test, NS, p = 0.5185, t = 0.7071, df = 4.000, n = 3 for each group) (**i**) and neuronal progeny (DCX^+^ cells, imaged: 508.0 ± 138.2 cells; control: 611.3 ± 87.6 cells; two-tailed unpaired t-test, NS, p = 0.3457, t = 0.1094, df = 3.385, n = 3 for each group) (**j**). DG, dentate gyrus, dpi, day-post-injection, IF, immunofluorescent, mpi, monnth-post-injection, NR cells, non-radial cells, NS, not significant, NSCs, neural stem cells, R cells, radial-like cells, TAM, tamoxifen and wpi, week-post-injection. All data were presented as mean ± SEM. Scale bars, 10 μm. NS p > 0.05, *p < 0.05, **p < 0.01, ***p < 0.001, ****p < 0.0001. For detailed statistics, see Supplementary Table 1. Source data

**Extended Data Fig. 2:**
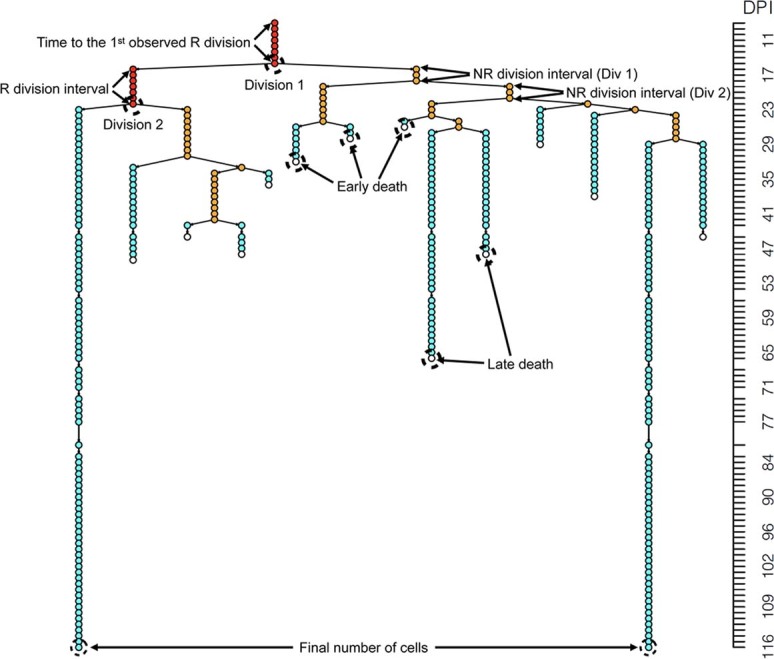
Schematic illustration of different parameters extracted from lineage data of imaged clones. Shown are the parameters used for the coding of clonal behaviors of NSCs (for experimental details refer to the Methods section).

We first analyzed NSC pool size, proliferation and neurogenic output using young (2MO) and middle-aged (12MO) Nestin-GFP mice (Fig. 1a) (ref. ^28^). Consistent with the results of previous studies^9,10,19,22^, the number of radial glia-like NSCs (hereafter called R cells) and nonradial glia-like progenitors (hereafter called NR cells) declined from 2MO to 12MO (Fig. 1b,c). Further, the population of proliferating NSCs declined, as measured by colabeling with cell cycle protein KI67 (Fig. 1d,e), causing a reduction with age in the number of newly generated neurons expressing doublecortin (DCX) (Fig. 1f,g) and corroborating previous findings that neurogenesis is strongly reduced in 12MO mice compared with young adult mice. Using intravital imaging, we aimed to identify the cellular principles mediating the observed age-dependent decline in hippocampal neurogenesis. We used in vivo two-photon microscopy to follow sparsely labeled R cells that were genetically targeted in Gli1-Cre^ERT2^::*Rosa26*-LSL-tdTomato (TOM) young (2MO) and middle-aged (12–14MO) mice upon injection of tamoxifen (TAM) (Extended Data Fig. 1a) (ref. ^5^). Starting R cells were identified by the presence of a radially oriented process that could be identified unambiguously (Supplementary Video 1) (refs. ^4,5^). Middle-aged mice received a higher dose of TAM (180 mg kg^−1^) than young mice, given the sparseness of remaining NSCs at this age (Fig. 1c), targeting approximately 20% of R cells in the middle-aged DG (Extended Data Fig. 1b,c) (ref. ^5^). Gli1-mediated recombination predominantly labeled R cells in the DG of middle-aged mice, resulting in longer chases after TAM injection in the generation of neuronal progeny, as expected (Extended Data Fig. 1d–f) (ref. ^5^). Implantation of a cortical window and repeated intravital imaging did not significantly affect the size of the NSC pool, or the proliferation or differentiation of NSCs in middle-aged mice, consistent with previous findings in young adult mice (Extended Data Fig. 1g–j) (refs. ^4,5^). Cellular dynamics and cell fate of individual clones were tracked for up to 115 days, using a previously established approach (Fig. 1h) (refs. ^4,5^). In total, we analyzed 47 active clones in 12–14MO mice and compared their behavior and clonal features with those of 56 tracked clones in 2MO mice that had been recorded previously under identical experimental conditions (Extended Data Fig. 2 and Supplementary Fig. 1) (ref. ^5^).

**Extended Data Fig. 3:**
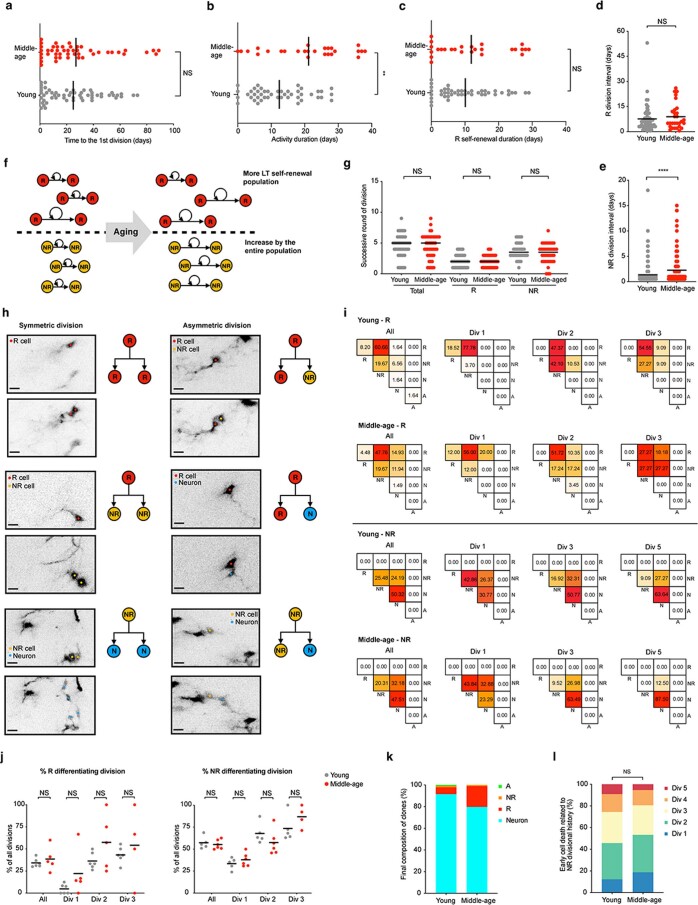
Proliferative capacity and modes of cell division are largely preserved at middle-age. **a**, Time until the first division of Gli1-targeted R cell between young and middle-aged lineages (young: 26.98 ± 24.70 days, n = 56 clones/6 mice; middle-age: 24.98 ± 20.79 days, n = 43 clones/5 mice; two-tailed Mann-Whitney test, NS, p = 0.1303, *U* = 1303). **b**, The activity duration of clones without long-term self-renewal R cells is elongated in the middle-aged lineages (young: 12.40 ± 8.29 days, n = 45 clones/6 mice; middle-age: 21.15 ± 11.64 days, n = 26 clones/5 mice; two-tailed Mann-Whitney test, *p = 0.0013, *U* = 321). **c**, The R self-renewal duration of R cells that are not long-term self-renewal shows no difference between young and middle-age lineages (young: 10.11 ± 8.40 days, n = 45 clones/6 mice; middle-age: 11.88 ± 10.49 days, n = 26 clones/5 mice; two-tailed Mann-Whitney test, NS, p = 0.6358, *U* = 545). **d-e**, The dividing intervals of R cells that are not long-term self-renewal and NR cells that are derived from not long-term self-renewal R cells. Average R dividing interval per clone (young: 7.63 ± 8.09 days, n = 56 divisions/6 mice; middle-age: 8.91 ± 7.60 days, n = 29 divisions/5 mice; two-tailed Mann-Whitney test, NS, p = 0.5001, *U* = 712) (**d**). Average NR dividing interval per clone (young: 1.36 ± 1.91 days, n = 260 divisions/6 mice; middle-age: 2.28 ± 2.71 days, n = 172 divisions/5 mice; two-tailed Mann-Whitney test, ****p < 0.0001, *U* = 20759) (**e**). **f**, Schematic illustration summaries that the elongation of dividing intervals of R cells mainly results from the increased proportion of long-term self-renewal R cells wheras the elongation of dividing intervals of NR cells is independent to the long-term self-renewal state of parent R cells. **g**, The successive rounds of division between two ages (Total division, young: 4.40 ± 1.92 rounds, n = 56 clones/6 mice; middle-age: 4.45 ± 1.79 rounds, n = 43 clones/5 mice; two-tailed Mann-Whitney test, NS, p = 0.9054, *U* = 1154; R division, young: 2.04 ± 0.93 rounds, middle-age: 2.19 ± 1.01 rounds, two-tailed Mann-Whitney test, NS, p = 0.9676, *U* = 1254; NR division, 3.29 ± 1.53 rounds, middle-age: 3.74 ± 1.27 rounds, two-tailed Mann-Whitney test, NS, p = 0.2895, *U* = 800). **h**, The classification of cell division modes. The symmetric division is defined as two daughter cells keep the same identity as the mother cell which can be either self-renewal division when one mother cell generates two daughters with the same identity or differentiation division when one mother cell generates two daughters with different identities. The asymmetric division is defined as two daughter cells have different identities which can be either self-renewal division when one daughter keeps the same identity as the mother cell or differentiation division when one mother cell generates two daughters with different identity. **i**, The division modes of R and NR cells are largely preserved during aging. (Upper) Heatmaps represent the frequencies of modes of R division (young: n = 61 of all divisions, n = 27 of Div1 divisions, n = 19 of Div2 divisions, n = 11 of Div3 divisions; middle-age: n = 67 of all divisions, n = 25 of Div1 divisions, n = 29 of Div2 divisions, n = 11 of Div3 divisions). (Lower) Heatmaps represent the frequencies of modes of NR division (young: n = 310 of all divisions, n = 91 of Div1 divisions, n = 65 of Div3 divisions, n = 11 of Div5 divisions; middle-age: n = 261 of all divisions, n = 73 of Div1 divisions, n = 63 of Div3 divisions, n = 8 of Div5 divisions). **j**, Quantification of consumptive division of R and NR cells that are differentiating between young and middle-aged lineages. (Left) Percentage of differentiated division of R cells (neither of two progeny cells is R cell) in both ages (All division, young: 34.18 ± 4.92%, 61 divisions/6 mice; middle-age: 38.66 ± 13.62%, 67 divisions/5 mice; two-tailed Mann-Whitney test, NS, p = 0.5671, *U* = 11.5; Div 1, young: 4.75 ± 5.46%, 27 divisions/6 mice; middle-age: 22.19 ± 25.70%, 25 divisions/5 mice; two-tailed Mann-Whitney test, NS, p = 0.0563, *U* = 4.5; Div 2, young: 36.14 ± 9.26%, 19 divisions/6 mice; middle-age: 57.58 ± 31.16%, 29 divisions/5 mice; two-tailed Mann-Whitney test, NS, p = 0.4004, *U* = 10; Div 3, young: 43.31 ± 9.20%, 11 divisions/6 mice; middle-age: 54.17 ± 41.67%, 11 divisions/5 mice; two-tailed Mann-Whitney test, NS, p = 0.4000, *U* = 7.5). (Right) Percentage of differentiated division of NR cells (neither of two progeny cells is NR cell) in both ages (All division, young: 57.20 ± 6.93%, 310 divisions/6 mice; middle-age: 55.20 ± 5.30%, 261 divisions/5 mice; two-tailed Mann-Whitney test, NS, p = 0.5368, *U* = 11; Div 1, young: 33.58 ± 6.47%, 91 divisions/6 mice; middle-age: 37.85 ± 7.48%, 73 divisions/5 mice; two-tailed Mann-Whitney test, NS, p = 0.4589, *U* = 10.5; Div 3, young: 67.76 ± 11.54%, 65 divisions/6 mice; middle-age: 57.45 ± 13.96%, 63 divisions/5 mice; two-tailed Mann-Whitney test, NS, p = 0.0823, *U* = 5; Div 5, young: 73.53 ± 15.24%, 11 divisions/4 mice; middle-age: 86.75 ± 12.33%, 8 divisions/3 mice; two-tailed Mann-Whitney test, NS, p = 0.2063, *U* = 4.5). **k**, Quantification of the final composition of active clones. Young: 6.30% R cells, 1.50% NR cells, 91.50% neurons and 0.70% astrocytes, n = 282 cells/6 mice; middle-age: 19.19% R cells, 1.01% NR cells and 79.80% neurons, no astrocyte, n = 99 cells/5 mice. **l**, The distribution of early cell death according to their divisional history (young: Div1 12.2%, Div2 33.5%, Div3 28.7%, Div4 16.5%, Div5 9.1%; middle-age: Div1 18.7%, Div2 34.6%, Div3 27.2%, Div4 14.1%, Div5 5.4%) with no statistical difference (Chi-square test, NS, p = 0.1840, Chi-square = 6.211, df = 4). Div, division, NS, not significant and NR cells, non-radial cells. All data are shown as mean ± SEM. Scale bars, 10 μm. NS p > 0.05, *p < 0.05, **p < 0.01, ***p < 0.001, ****p < 0.0001. For detailed statistics, see Supplementary Table 1. Source data

Whereas the majority of Gli1-targeted R cells remained quiescent at all ages, the fraction of R cells recruited into the proliferative pool was substantially reduced in 12–14MO mice compared with 2MO mice, in line with the results of our Nestin-GFP experiments, indicating reduced activation of R cells in mice of advanced age (Fig. 1i) (refs. ^16,19,23,29^). When R cells entered the cell cycle, they showed no substantial differences in the time to first cell division in young compared with middle-aged mice (Fig. 1j,k and Extended Data Fig. 3a). We next analyzed the clonal output, the number of newborn cells generated, of individual active R cells that became active during the time course. Strikingly, we found that the final number of cells generated within clones (clone size) was markedly reduced in 12–14MO mice compared with 2MO mice (Fig. 1l), indicating that cellular output of individual R cells is reduced with age, a finding supported by recent static lineage tracing experiments^29^.

## Cellular dynamics of middle-aged NSCs

**Fig. 2:**
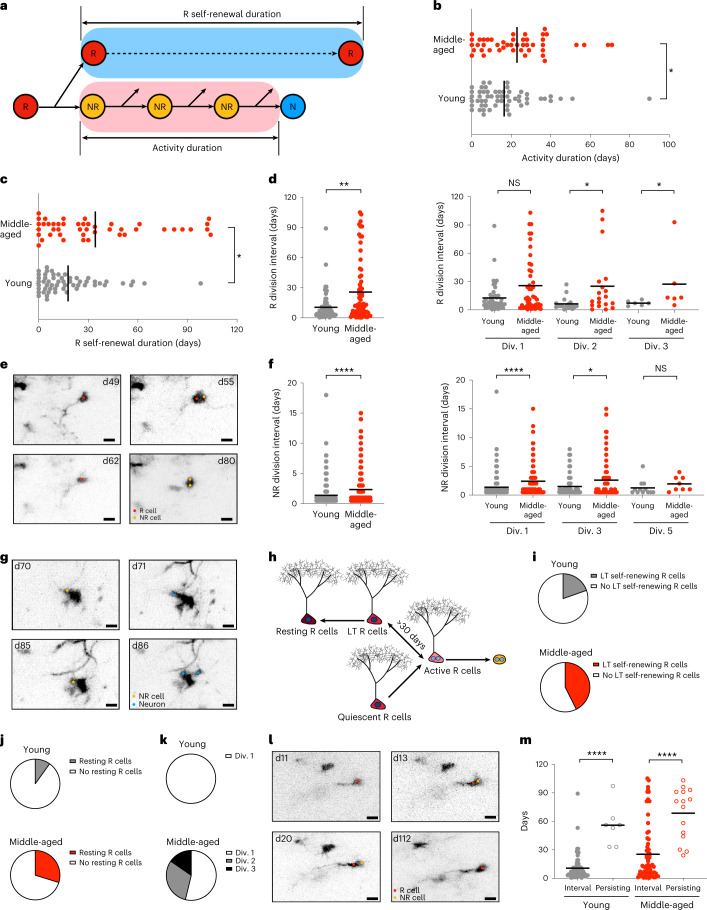
Cell cycle kinetics are slowed down and a substantial proportion of active NSCs return to long-term quiescence at middle age. **a**, Pictogram illustrating the definition of activity duration and R self-renewal duration. **b**, Activity duration is elongated in middle-aged lineages (young: 16.39 ± 15.81 days, *n* = 56 clones from six mice; middle-aged: 22.55 ± 17.34 days, *n* = 47 clones in five mice; two-tailed Mann–Whitney test, *P = 0.0361, *U* = 954). **c**, R self-renewal duration is elongated in middle-aged lineages (young: 17.80 ± 19.31 days, *n* = 56 clones from six mice; middle-aged: 34.02 ± 32.26 days, *n* = 47 clones from five mice; two-tailed Mann–Whitney test, *P = 0.0139, *U* = 902). **d**, Dividing intervals of R cells. Left: average R dividing interval per clone (young: 10.48 ± 13.43 days, *n* = 61 divisions from six mice; middle-aged: 25.68 ± 30.21 days, *n* = 67 divisions from five mice; two-tailed Mann–Whitney test, **P = 0.0049, *U* = 1695). Right: R dividing interval in successive rounds of division (Div. 1: young 12.64 ± 15.85 days, *n* = 27 divisions from six mice; middle-aged 25.70 ± 29.55 days, *n* = 25 divisions from five mice; two-tailed Mann–Whitney test, NS, *P* = 0.2434, *U* = 789; Div. 2: young 6.42 ± 6.67 days, *n* = 19 divisions from six mice; middle-aged 25.11 ± 32.54 days, *n* = 29 divisions from five mice; two-tailed Mann–Whitney test, *P = 0.0138, *U* = 91; Div. 3: young 7.17 ± 2.56 days, *n* = 11 divisions from six mice; middle-aged 27.33 ± 33.04 days, *n* = 11 divisions from five mice; two-tailed Mann–Whitney test, *P = 0.0281, *U* = 4.5). **e**, Representative images of an active R cell in middle-aged mice undergoing two successive rounds of division with relatively long dividing intervals (25 days). **f**, Dividing intervals of NR cells. Left: average NR dividing interval per clone (young: 1.38 ± 1.83 days, *n* = 310 divisions from six mice; middle-aged: 2.33 ± 2.82 days, *n* = 261 divisions from five mice; two-tailed Mann–Whitney test, ****P < 0.0001, *U* = 56841). Right: NR dividing interval in successive divisions (Div. 1: young 1.37 ± 2.03 days, *n* = 91 divisions from six mice; middle-aged 2.41 ± 2.76 days, *n* = 73 divisions from five mice; two-tailed Mann–Whitney test, ****P < 0.0001, *U* = 4669; Div. 3: young 1.50 ± 1.83 days, *n* = 65 divisions from six mice; middle-aged 2.59 ± 3.32 days, *n* = 63 divisions from five mice; two-tailed Mann–Whitney test, *P = 0.0249, *U* = 2667; Div. 5: young 1.25 ± 1.31 days, *n* = 11 divisions from four mice; middle-aged 1.94 ± 1.27 days, *n* = 8 divisions from three mice; two-tailed Mann–Whitney test, NS, *P* = 0.1120, *U* = 27.5). **g**, Representative images of an active NR cell in middle-aged mice undergoing two successive rounds of division with relatively long dividing intervals. **h**, Long-term (LT) self-renewal of an R cell is defined as return to quiescence after proliferation for >30 days. Resting R cells are defined as those long-term self-renewing R cells that did not divide again after return to quiescence. **i**, Pie charts showing proportions of long-term self-renewing R cells in active lineages (young: 19.64%, *n* = 11; middle-aged: 42.55%, *n* = 20). **j**, Pie charts showing proportions of resting R cells in active lineages (young: 8.92%, *n* = 5; middle-aged: 29.79%, *n* = 14). **k**, Pie charts showing proportions of resting R cells according to their divisional history (young: Div. 1, 100%; middle-aged: Div. 1, 53.80%, Div. 2, 30.80%, Div. 3, 15.40%). **l**, Representative images of a resting R cell in the aged lineage after two rounds of activation. **m**, Comparison of R dividing intervals and persisting time of resting R cells, showing that the persisting time of resting R cells is substantially longer than the R cell division interval (young: R division interval 10.48 ± 13.73 days, *n* = 67 divisions from six mice versus persisting time 55.86 ± 21.59 days, *n* = 7 cells from six mice, two-tailed Mann–Whitney test, ****P < 0.0001, *U* = 8.5; middle-aged: R division interval 25.68 ± 29.98 days, *n* = 70 divisions from five mice versus persisting time 68.40 ± 27.20 days, *n* = 15 cells from five mice, two-tailed Mann–Whitney test, ****P < 0.0001, *U* = 144.5). Div., division. All data are presented as mean ± s.e.m. Scale bars, 10 μm. NS, *P* > 0.05, *P < 0.05, **P < 0.01, ***P < 0.001, ****P < 0.0001. For detailed statistics, see Supplementary Table 1Source Data. Source Data

To identify a potential cause for reduced output of R cells with advancing age, we analyzed kinetics of cell divisions and fate behavior of neurogenic cells. Total duration of activity (that is, time from the first observed cell division to the last division of any proliferative cell in the clone) and total R self-renewal duration (that is, time from the first R division to the last point the active R cell was observed) were increased with age (Fig. 2a–c and Extended Data Fig. 2). Further, the interval between cell divisions of R and NR cells appeared to be longer in advanced age precursors when all clones recorded were analyzed (Fig. 2d–g), suggesting that age-related alterations of neurogenic cells may be influenced by the individual division history of the cell^23,30^. However, extended division intervals in R cells but not in NR cells were influenced by the relative proportion of cells that showed long-term self-renewal (>30 days), which increased in middle-aged mice compared with young mice (Fig. 2h–i and Extended Data Fig. 3b–e). We next analyzed whether cell division capacity and cell fate choices of hippocampal precursors were affected by advancing age. Successive rounds of cell divisions in 2MO and 12–14MO mice were comparable for R and NR cells (Extended Data Fig. 3g), suggesting that the potential for cell division of hippocampal precursors is not affected by advancing age. Cell division modes were classified using classic categorizations of progenitor cell divisions (Extended Data Fig. 3h) (refs. ^4,5,22,31^). Modes of cell division were comparable in 2MO and 12–14MO mice (Extended Data Fig. 3h–i), suggesting that a developmental-like program, for example, sequentially moving from self-renewing divisions to more differentiating divisions^4^, is preserved with advancing age in neurogenic cells of the hippocampus. However, R cells in middle-aged mice showed distinct behavior in terms of long-term self-renewal (defined as return to quiescence for >30 days after proliferation) (Fig. 2h) (ref. ^5^). We found an increase in the proportion of activated R cells returning to long-term quiescence in middle-aged mice (Fig. 2i), corroborating previous results based on static lineage tracing experiments^23,29,32^. A fraction of long-term self-renewing R cells did not divide again but persisted throughout the observed period; these are referred to as resting R cells^20,23^. Indeed, the proportion of resting R cells was threefold higher in aged animals (Fig. 2j), consistent with an increased proportion of R cells in the final composition of clones with advancing age (Extended Data Fig. 3k). Resting R cells in young mice underwent strictly one round of division, whereas resting R cells in middle-aged mice underwent up to three cell divisions before they returned to long-term quiescence (Fig. 2k,l). Importantly, the persisting time of resting R cells was substantially longer than the R cell division interval, clearly indicating a return to long-term quiescence instead of extended division intervals going beyond the observation periods (Fig. 2m). We further compared the distribution of individual values of the R cell division intervals and the time to the first cell division using Kolmogorov–Smirnov testing. Indeed, the time to first cell division and observed division intervals were significantly distinct for R cells in young mice (young: *P* < 0.0001, Kolmogorov–Smirnov *D* = 0.4725; middle-aged: *P* > 0.05, Kolmogorov–Smirnov *D* = 0.1733), suggesting that R cells in young mice had not divided immediately before the onset of imaging (Fig. 2d and Extended Data Fig. 3a). However, we found no significant difference between division interval and time to first division for R cells in middle-aged mice (*P* > 0.05), indicating that some R cells might have had divisional history before the onset of the imaging experiments (Fig. 2d and Extended Data Fig. 3a). Taken together, these results support the concept that once activated, a fraction of young R cells undergoes a burst of divisions and becomes depleted within a relatively short time; by contrast, R cells in middle-aged mice appear to return to longer term quiescence and reenter the cell cycle at later points^23,32^.

## Cell death of neural progeny in middle-aged mice

**Fig. 3:**
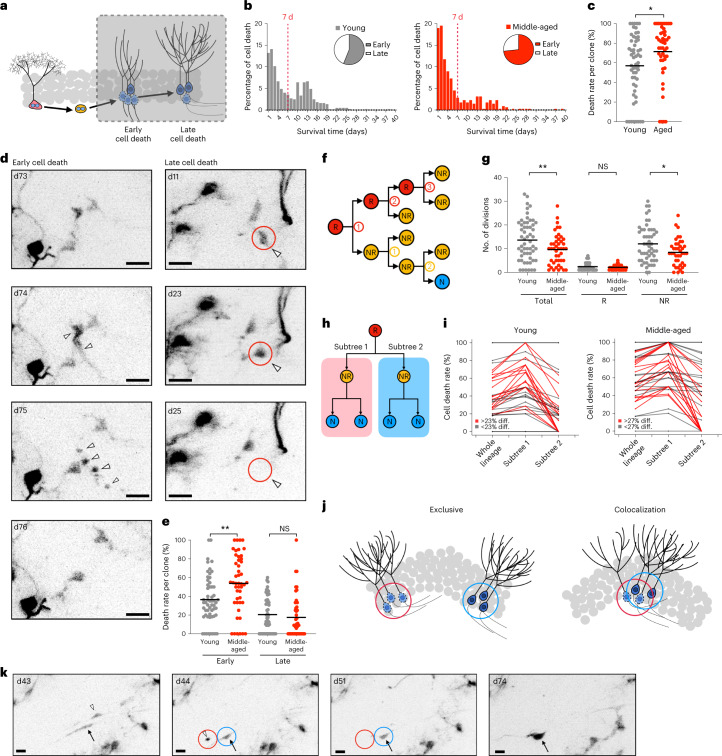
Survival of early neural progeny is reduced at middle age. **a**, Schematic of two typical waves of cell death occurring before and after 7 days after birth. **b**, Both waves were observed in both young (left) and middle-aged (right) mice (young: *n* = 456 events in six mice; middle-aged: *n* = 360 events in five mice). The relative percentages of early and late cell death in young and middle-aged mice are embedded in the corresponding histogram (young: early 56.14% and late 43.86%; middle-aged: early 73.33% and late 26.67%). **c**, Comparison of total cell death rates at both ages. Aged lineages displayed higher total death rate compared with their younger counterparts (young: 57.03 ± 31.73%, *n* = 56 clones, 456 events in six mice; middle-aged: 71.40 ± 28.64%, *n* = 47 clones, 360 events in five mice; two-tailed Mann–Whitney test, *P = 0.0106, *U* = 932.5). **d**, Representative images of cell death events. Upper: example of early cell death 1 day after birth. Upper-middle and right: dying cells are labeled with an arrowhead. Lower: an example of late cell death 14 days after birth. The dying cell labeled by an arrowhead survived for 14 days until death. **e**, Comparison of early and late cell death rates at both ages. Early cell death rate was elevated in the middle-aged lineages (young: 36.55 ± 24.77%, *n* = 256 events in six mice; middle-aged: 53.92 ± 30.50%, *n* = 264 events in five mice; two-tailed Mann–Whitney test, **P = 0.0016, *U* = 845.5), whereas late cell death rate was comparable between the two ages (young: 20.48 ± 18.88%, *n* = 200 events in six mice; middle-aged: 17.48 ± 22.36%, *n* = 96 events in five mice; two-tailed Mann–Whitney test, NS, *P* = 0.2366, *U* = 1141). **f**, Schematic showing the quantification of number of cell divisions for R and NR cells. **g**, Total number of cell divisions in the active clones was reduced in the middle-aged lineages (total division: young 13.61 ± 8.60 divisions, *n* = 371 divisions in six mice; middle-aged 9.57 ± 6.43 divisions, *n* = 328 divisions in five mice; two-tailed Mann–Whitney test, *P = 0.0221, *U* = 972; R division: young 3.39 ± 2.07 divisions, *n* = 61 divisions in six mice; middle-aged 2.09 ± 1.00 divisions, *n* = 67 divisions in five mice; two-tailed Mann–Whitney test, NS, *P* = 0.5613, *U* = 1178; NR division: young 11.00 ± 6.86 divisions, *n* = 310 divisions in six mice; middle-aged 8.38 ± 5.57 divisions, *n* = 261 divisions in five mice; two-tailed Mann–Whitney test, *P = 0.0363, *U* = 775). **h**, Pictogram illustrating the comparison of cell death frequencies in subtree 1 and subtree 2 in comparison with the whole lineage. **i**, Differences in cell death frequencies in subtree 1 and subtree 2 displayed similar patterns between young and aged lineages. Differences in cell death between the two subtrees >23% (young) or 27% (middle-aged) are highlighted in red. **j**, Surviving and dying cells could mutually be exclusive with or overlap with each other. **k**, Representative images showing spatial overlap of surviving (shown by arrow in the blue circle) and dying (shown by open arrowhead in the red circle) cells. All data are shown as mean ± s.e.m. Scale bars, 10 μm. diff., difference. NS, *P* > 0.05, *P< 0.05, **P < 0.01, ***P< 0.001, ****P < 0.0001. For detailed statistics, see Supplementary Table 1Source Data. Source Data

Given that the division capacity of active R cells remained rather stable in middle-aged mice, we next analyzed cell death of R cell progeny with the aim of identifying the cause of the reduction in clone size in mice with advancing age. Consistent with previous reports^4,33^, we found two waves of cell death at both ages: an early phase, defined as cell death within 7 days after cell birth, and a late phase beyond 7 days (Fig. 3a). Average cell death was substantially increased in lineages observed in middle-aged mice (Fig. 3b), owing to a selective increase in early cell death rate (Fig. 3c,d). Indeed, enhanced cell death of early progeny caused a reduction in the total number of neurogenic cell divisions in individual clones, thereby mediating the final reduction of clone size with advancing age (Fig. 3e). This was supported by the finding that the distribution of early cell death according to divisional history was comparable between young and middle-aged lineages, indicating increased loss of NR cells by early cell death (Extended Data Fig. 3l). Intraclonal variability of early cell death among individual sublineages (Fig. 3f–i) and substantial spatial overlap of dying and surviving cells appeared to be comparable between 2MO and 12–14MO mice (Fig. 3j,k) (ref. ^4^). In contrast to previous reports using static analyses combined with modeling approaches^19^, death of dormant R cells was extremely rare in both age groups (0 of 80 and 1 of 161 recorded dormant R cells died in young and middle-aged mice, respectively). Taken together, these findings indicate that early death of R cell progeny mediates the reduction in clone size of active stem cells with advancing age.

## Maturation of newborn neurons in middle-aged mice

**Fig. 4:**
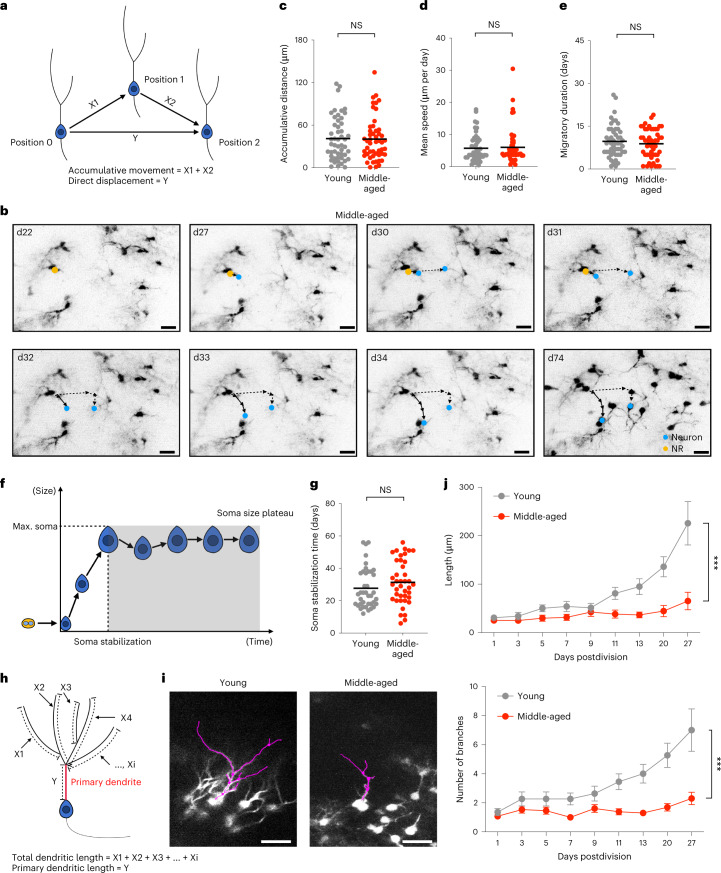
Heterogeneous behaviors of postmitotic progeny at middle age. **a**, The accumulative migratory distance is defined as the sum of each migratory distance, whereas the direct migratory displacement is defined as the distance between the starting and the last position of newly born granule cells. **b**, Representative images showing migratory trajectories of two granule cells (shown by arrows with solid lines and arrows with dashed lines, respectively) in middle-aged mice. **c**–**e**, Quantitation of the accumulative distance (young: 40.98 ± 30.57 μm, *n* = 40 neurons in three mice; middle-aged: 40.36 ± 30.47 μm, *n* = 40 neurons in five mice; two-tailed unpaired *t* test with Welch’s correction, NS, *P* = 9198, *t* = 0.1009, d.f. = 98) (**c**), mean migratory speed (young: 5.76 ± 6.02 μm per day, *n* = 40 neurons in three mice; middle-aged: 6.02 ± 5.61 μm per day, *n* = 40 neurons in five mice; two-tailed unpaired *t* test with Welch’s correction, NS, *P* = 0.7923, *t* = 0.2640, d.f. = 90.48) (**d**) and time of migration (young: 9.68 ± 5.66 days, *n* = 40 neurons in three mice; middle-aged: 8.86 ± 5.08 days, *n* = 40 neurons in five mice; two-tailed unpaired *t* test with Welch’s correction, NS, *P* = 0.4477, *t* = 0.7623, d.f. = 96.89) (**e**) between young and middle-aged newly born granule cells. **f**, Growing kinetics of granule cell soma, measured as the time when newly born granule cells reach the maximum size of the soma. **g**, Quantitative analysis of the time reaching the maximum size of the soma between young and middle-aged newly born granule cells (young: 27.69 ± 12.48 days, *n* = 40 neurons in three mice; middle-aged: 31.36 ± 13.52 days, *n* = 40 neurons in five mice; two-tailed unpaired *t* test with Welch’s correction, NS, *P* = 0.2119, *t* = 1.259, d.f. = 76.77). **h**, Graphical illustration of the measurement of the maturation process of newly born granule cells in terms of total length of dendrites, length of the primary dendrite, length of the longest branch and number of branches. **i**, Representative images of the measurement of dendrites of newly born granule cells in young and middle-aged mice. **j**, Quantitation of the total length of dendrites (young, *n* = 11 neurons in two mice; middle-aged, *n* = 13 neurons in five mice; two-way ANOVA, ***P = 0.0001, *F*(1.856, 42.69) = 11.89) and number of branches (young, *n* = 11 neurons in two mice; middle-aged, *n* = 13 neurons in five mice; two-way ANOVA, ***P = 0.0004, *F*(1.958, 45.03) = 9.408) of newly born granule cells in the first 28 days after birth. All data are presented as mean ± s.e.m. Scale bars, 20 μm. Max., maximum. NS, *P* > 0.05, *P < 0.05, **P < 0.01, ***P < 0.001, ****P < 0.0001. For detailed statistics, see Supplementary Table 1Source Data. Source Data

**Extended Data Fig. 4:**
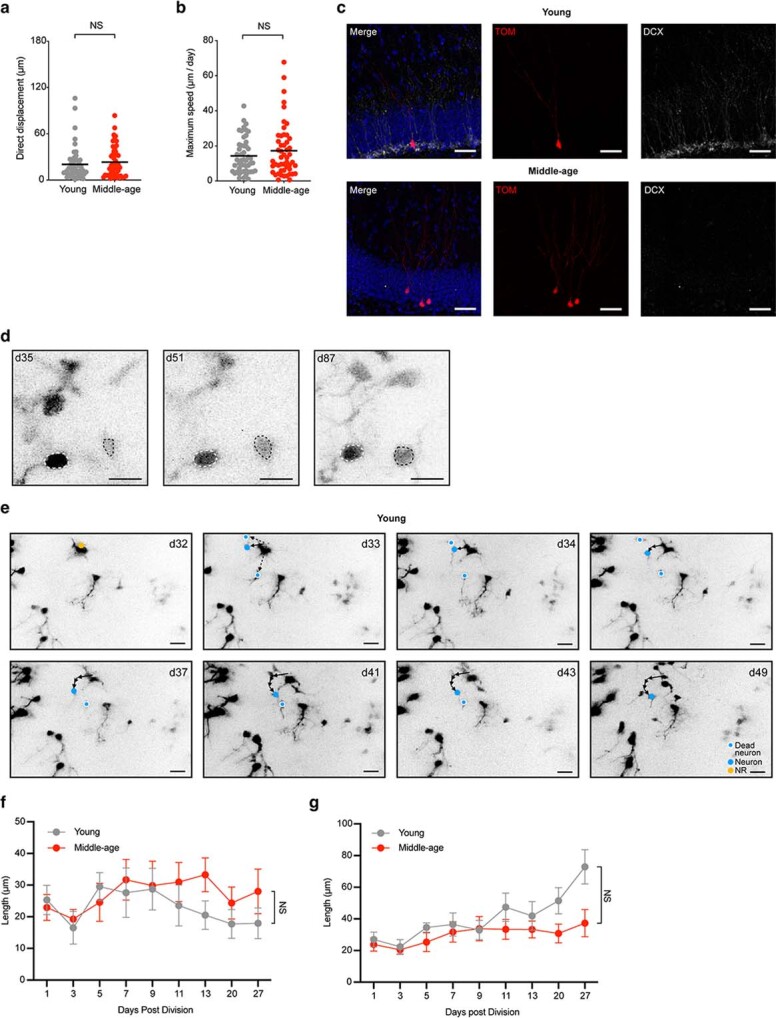
Migration of newborn neurons in mice at middle-age shows similar patterns compared to young mice. **a-b**, Quantitation shows no significant difference in terms of the direct migratory displacement (young: 20.39 ± 21.28 μm, n = 40 neurons/3 mice; middle-age: 23.27 ± 19.68 μm, n = 40 neurons/5 mice; two-tailed unpaired t-test, NS, p = 0.4848, t = 0.7013, df = 97.41) (**a**) and maximum migratory speed (young: 14.28 ± 10.05 μm/day, n = 40 neurons/3 mice; middle-age: 17.25 ± 15.13 μm/day, n = 40 neurons/5 mice; two-tailed unpaired t-test, NS, p = 0.2504, t = 1.157, df = 85.19) (**b**) of newly born granule cells between two aged. **c**, Representative immunofluorescence images showing leaky granule cells presenting in the subgranular zone shortly after TAM induction (2dpi) in both young and middle-aged mice that are lack of newly born neuronal marker (Dcx). **d**, Representative images showing the measurement of the soma size of newly born granule cells (black dashed circle). The value was normalized by the value of another mature granule cell (white dashed circle) in the same spot. **e**, Representative images showing migratory trajectories of one granule cell in the young mice. Leaky granule cells can be observed in the same image. **f-g**, Quantitation shows no significant difference in terms of the length of primary dendrite (young, n = 11 neurons/2 mice; middle-age, n = 13 neurons/5 mice; two-way ANOVA, NS, p = 0.2391, F(3.137, 72.16) = 1.433) (**f**) and the length of the longest branch (young, n = 11 neurons/2 mice; middle-age, n = 13 neurons/5 mice; two-way ANOVA, NS, p = 0.1850, F(8, 198) = 8.432) (**g**) in two ages. All data are shown as mean ± SEM. Scale bars, 20 μm. NS p > 0.05, *p < 0.05, **p < 0.01, ***p < 0.001, ****p < 0.0001. For detailed statistics, see Supplementary Table 1. Source data

To study whether migratory behavior was affected in middle-aged mice, we measured the speed and total migration of individual newborn granule cells (Fig. 4a,b and Supplementary Video 3). Neither the migratory distances nor the migratory speed of newborn granule cells showed significant differences between newborn neuronal daughter cells recorded in 2MO and 12–14MO mice (Fig. 4c,d, and Extended Data Fig. 4a,b). Further, the total duration of migration was comparable between the two age groups (Fig. 4e).

Snapshot-based analyses of newborn granule cells have suggested that the rate of neuronal maturation on a population level decreases with advancing age^34,35^. Intravital imaging allows neuronal maturation to be followed at the single-cell level with high temporal resolution to study the effects of advancing age on granule cell maturation. Notably, newborn granule cells showed heterogeneous changes with advanced age. Whereas we did not observe altered kinetics of cell soma growth (Fig. 4f,g and Extended Data Fig. 4c,d), we found delayed maturation of newborn granule cells in middle-aged mice in terms of the total length of extending dendrites and the number of dendritic branches within the first 4 weeks after birth (Fig. 4h,j, Extended Data Fig. 4e and Supplementary Video 4), consistent with previous reports^34,35^. These results indicate heterogeneity of age-related changes in the maturation of postmitotic neuronal progeny with age and suggest that maturation is not globally delayed with advancing age; rather, dendritic growth and relatively late-occurring morphological complexity are affected.

**Extended Data Fig. 5:**
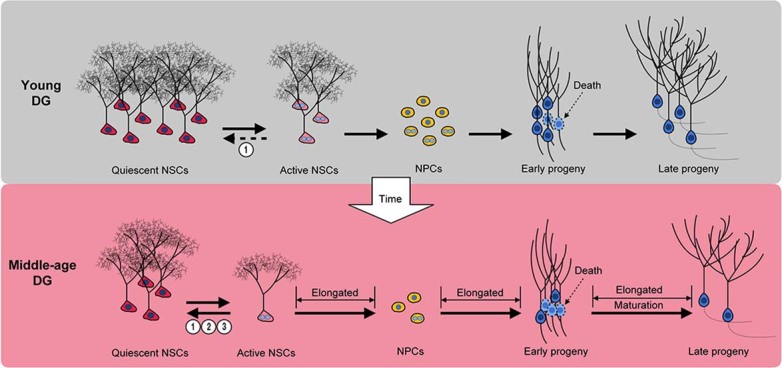
Summary of age-related changes of mouse hippocampal NSCs identified through intravital imaging. We summarized the age-related changes of adult mouse hippocampal NSCs from the activation of R cells and proliferation of NSCs to the survival and maturation of their neuronal progeny.

Previous studies largely relied on static pulse-chase lineage tracing assays to recover lineage information of neurogenic cells in the aging hippocampus, yielding ambiguous and uncertain results owing to limited temporal resolution and the inability to resolve the dynamics of individual lineages^23,29,32^. Using chronic intravital imaging, we here define changes that occur with advancing age in hippocampal stem cells and their progeny in middle-aged mice (12–14MO), a time in the lifespan where neurogenesis has already dramatically declined^1^. We show that (1) NSCs show reduced proliferative activity with age, causing reduced numbers of R cells to become active; (2) with advancing age, a fraction of R cells shows extended self-renewal duration; (3) the age-associated reduction in clonal output of R cells is largely due to increased early cell death, whereas the cell division capacity of progenitors is not substantially altered with age; and (4) advancing age causes maturational delays of individual newborn neurons (Extended Data Fig. 5). However, future work will need to extend intravital recordings of NSCs and their progeny in aged mice (≥24MO), which is currently technically not feasible, to identify potential additional alterations in neurogenic lineage characteristics in aged mice compared with young and middle-aged mice. Furthermore, repetitive anesthesia, as used here for imaging sessions, may affect the behavior of NSCs in young and middle-aged mice; future work could use awake mice that are habituated to head fixation, even though such an approach may reduce image quality owing to motion artefacts.

Together with previous work suggesting that a substantial number of NSC divisions are consumptive early in adulthood, most likely being responsible for the diminished NSC pool in middle-aged mice^4,5,23,29,32^, the data presented here identify early cell death as a key mechanism that causes reduced clonal output of R cells with advancing age. Further, our data suggest that neurogenic cell divisions of Gli1-targeted NSCs become less consumptive and that R cells in middle-aged mice much more frequently return to a resting state, a finding that is in line with previous static, snapshot-based data^32^. Thus, future attempts to enhance neurogenesis in mice with age-related decline of neurogenesis should not only aim to alter the cellular fate or division capacity of neurogenic precursors but also attempt to reduce early cell death of stem cell progeny^24–26^. Indeed, the molecular mechanisms underlying the early wave of cell death upon stem cell activation remain largely unknown. Analysis of the transcriptomes of dying cells^36^, use of other single-cell RNA sequencing-based techniques including spatial transcriptomics^37^ and protein-based analyses of the aging niche^38^ will be required to understand the molecular mechanisms that ultimately cause the age-related decline in neurogenesis. The data shown here define the cellular principles associated with reduced neurogenesis in the mouse hippocampus with advancing age using chronic intravital imaging.

## Methods

### Transgenic animals and TAM administration

All animals were group-housed on a 12-h light/dark cycle with ad libitum access to food and water. The Gli1-Cre^ERT2^::*Rosa26*-LSL-tdTomato mouse line (Gli1-Cre^+/−^Ai14^+/−^) was generated by crossing Gli1-Cre^ERT2^ mice (Gli1^tm3(cre/ERT2)Alj^; the Jackson Laboratory, 007913) and the CAG tdTomato (Ai14;B6.Cg-Gt(ROSA)^26Sortm14(CAG-tdTomato)Hze^; the Jackson Laboratory, 007914) reporter line as described previously^5^. The Nestin-EGFP (B6.Cg-Tg(Nes-EGFP)1Yamm/Rbrc) mouse line was as described previously^28^. For intravital imaging of young (2MO) mice, data were collected from previous work^5^. For intravital imaging of middle-aged (12–14MO), Gli1-Cre^ERT2^ mice of mixed sex were used. A single intraperitoneal (i.p.) injection of TAM, 70–80 mg per kg body weight (Sigma), allowing for sparse labeling of R cells in 2MO mice was as used before^5^. Given the reduced numbers of NSCs in middle-aged mice, we increased the dose of TAM to 180 mg per kg body weight, resulting in an average number of 436 recombined cells in the subgranular zones of these mice (*n* = 4) at 2 days postinjection (dpi) (Extended Data Fig. 1a,b). The majority of TOM^+^ cells were R cells, based on their morphology and the expression of stem cell markers (GFAP and Sox2) and lack of astrocytic marker S100b (Extended Data Fig. 1a,b), representing up to 22% of all NSCs in the middle-aged mouse DG (Extended Data Fig. 1c). The fractions of R cells and newborn neurons at different time points after TAM injection are shown in Extended Data Fig. 1d,e. All animal experiments were approved by the Cantonal Commission for Animal Experimentation of the Canton of Zurich, Switzerland, in accordance with national and cantonal regulations and performed in accordance with the guidelines.

### Chronic intravital hippocampal imaging window implantation

The implantation of the hippocampal imaging window was carried out in mice aged 12–14 months as explained in previous reports^4,5^. Briefly, mice were deeply anesthetized with isoflurane (2% for induction and 1.5% for maintenance) and provided with analgesia (buprenorphine). The skin was opened, and the cranial bone was exposed and locally removed above the dorsal DG (− 2.0 mm posteriorly and −1.5 mm laterally from the bregma, 3 mm in diameter). The cortical tissue above the level of the corpus callosum (3 mm in diameter and 1.5 mm in depth) was then removed sequentially using a biopsy punch (Miltex) and a blunt 22-gauge needle for aspiration. The hippocampal imaging window (stainless steel cannula, 3 mm in diameter and 1.5 mm in height, covered by a glass coverslip; Warner Instruments) was inserted and stabilized in place using a stereotactic arm and stably fixed to the cranial bone with ultraviolet-cured dental cement (Ivoclar Vivadent) when bleeding stopped.

### Intravital two-photon imaging of hippocampal NSCs

Chronic intravital two-photon imaging was performed from 2 to 3 weeks after hippocampal window surgery and 2 days after TAM administration, as described previously^4,5^. Briefly, an aluminum headpost was added onto the contralateral side of the mouse head using ultraviolet-cured dental cement (Ivoclar Vivadent) to stabilize the mice during imaging experiments. Mice were deeply anesthetized with isoflurane (2% for induction and 1.5% for maintenance), while their body temperature was monitored and maintained using a heating pad at 37 °C. The imaging experiments were performed on a custom-built two-photon microscope (Movable Objective Microscope; Sutter Instrument) using a long-working-distance objective (water immersion, 16× magnification and 0.8 NA; Nikon), equipped with a Pockels cell (model 350/80 with controller model 302RM; Conoptics) and galvanometric scan mirrors (model 6210; Cambridge Technology), controlled by Helioscan software (https://github.com/HelioScan/HelioScan). The excitation of tdTomato was performed with a fiber oscillator laser at 1,070 nm (Fidelity-2; Coherent) or an ytterbium-doped laser system at 1,045 nm with 200 fs pulse width (High-Q lasers; FemtoTrain) to excite tdTomato-labeled cells in the DG. Emission signals were detected using a photomultiplier tube (Hamamatsu) after passing a red emission filter (610/75 nm; AHF). The lower part of the rim of the imaging cannula was used as the coordinate landmark to enable revisiting of the same imaging field of view (hereafter referred to as a SPOT) in subsequent imaging sessions and to perform clonal tracking (zero position: *x* = 0, *y* = 0 and *z* = 0). SPOTs containing identified R cells were selected for the subsequent imaging sessions, and around ten SPOTs were obtained for each animal. Individual SPOTs were selected based on two criteria: (1) containing an unambiguously identified single R cell; and (2) ensuring a positioning allowing for a total duration of imaging per mouse of less than 1 h per session. Each SPOT was imaged repeatedly by acquisition of a z-stack (512 × 512-pixel resolution, 2× zoom and 5 μm step-size), carefully considering and checking for all cells of the clone. All SPOTs were checked daily and scanned unless no changes occurred. The duration of the imaging session was minimized (<45 min per day). The following experimental settings remained identical for 2MO^5^ and 12–14MO mice: hippocampal window surgery, identification of R-cell-containing SPOTs, microscopy hardware and software settings, and data analyses. The numbers of z-stacks are detailed in Supplementary Table 2.

### Identification and coding of lineages after processing raw imaging data

The detailed processing and coding of imaging data were as described previously^4,5^. Briefly, all time points and *z* planes of chronically recorded SPOTs were compiled into a single Image5D file using a custom script. The key information in terms of morphology, dynamic morphological changes, cell division, migration and cell death from all *z* levels was taken into account to identify cell types, cellular behavior and lineage relationships. The function ‘ROI manager’ in FIJI (v.2.9.2) was used to code each individual cell at every time point throughout the compiled imaging file to serve as input for further processing in R. The coding parameters included: CellID, CellType, Uncertainty CellType, Timepoint, MotherID, Uncertainty MotherCell, SisterID, Uncertainty SisterCell and CellDeath. Only lineages starting with one R cell or one R cell with one daughter cell were included, as the first division of an R cell is critical for NSC behaviors. Each lineage was annotated with a code by two researchers independently and required agreement from both researchers.

### Lineage tree coding and analyses

The region of interest (ROI) results generated in the previous step were further processed in R (v.3.6.3) to assemble the lineage tree and for detailed data analysis using custom scripts as described previously^4,5^. Fifty-six active clones from young mice were taken from our previous work^5^. Two independent researchers went through all the coding to ensure the coding of the entire dataset was done under the same standard. In total, the dataset consisted of 103 (56 young and 47 aged) active clones.

Multiple parameters were used to describe the behavior of the active clones^4,5^. The values of all parameters were analyzed using the R script and then double-checked and corrected manually, except the activation rate of all imaged clones. The following parameters were used.

- Final cell number: the number of cells in the active clone at the last time point of the imaging experiment (Fig. 1l and Extended Data Fig. 2).
- Final cell composition: the composition of cell types in the active clone at the last time point of the imaging experiment (Extended Data Fig. 3f).
- Numbers of total, R and NR successive divisions: the maximum numbers of rounds of successive divisions in the same active clone (both R and NR cells were considered). Only CERTAIN (see below) R and NR cells were considered (Extended Data Figs. 2 and 3b).
- Activity duration of the clone: the time (d) from the first R division to the last division of any progenitor cell (R or NR) in the clone. If the clone only divided once, the activity duration was 0. Only CERTAIN R and NR cells were considered (Fig. 2a,b).
- R self-renewal duration: the time (d) from the first R division until the last time point at which the R cell was observed. If the R cell disappeared after the first division, the self-renewal time was 0. Only CERTAIN R cells were considered (Fig. 2a,c).
- Time between R and NR divisions: the time (d) between each R and NR divisions in the clone. For R cells, the first R root cell was excluded (which we defined as the time until the first division of the R cell) and all certain R cells were considered. If the R or NR cell was depleted after the first division, the time between R or NR divisions was 0. Only CERTAIN R and NR cells were considered (Fig. 2d,f and Extended Data Fig. 2).
- Time until the first observed division of the R cell: the time (d) from the beginning of TAM induction to the first R cell division observed. If the clone was observed with two cells at the first imaging session, a value of 1 was assigned (Extended Data Figs. 2 and 3a).
- Numbers of total, R and NR cell divisions: the total numbers of cell divisions in the clone. Only CERTAIN R and NR cells were considered (Extended Data Figs. 2 and 3b).
- Cell death: defined either by the disappearance of cells or debris of cell body being observed. The data were recorded as both numbers and percentages. The percentage of cell death was defined as the ratio of the number of dying cells to the number of total cell divisions. Early cell death was defined when cell death occurred within 7 days after birth. Late cell death was defined when cell death occurred beyond 7 days after birth. For cell death in subtrees, only cases where the main tree of the clone generated at least four terminal cells were considered. For early cell death according to divisional history, the rank of cell division of dying cells was extracted (Fig. 3 and Extended Data Figs. 2 and 3g). Given the size of the imaging field of view and the maximum recorded migratory speed of neural progeny per day (Fig. 4d and Extended Data Fig. 4b), together with the daily frequency of imaging, loss of cells due to migration out of the imaging field was extremely unlikely.

The criterion of certainty (CERTAIN or UNCERTAIN) of R and NR cells was based on whether two investigators (Y.W. and S.B.) reported nonambiguous or ambiguous cellular phenotypes during their independent coding of cells. If coding resulted in an ambiguous cellular phenotype, the phenotype was labeled as UNCERTAIN. CERTAIN R cells were unambiguously identified by two investigators as R cells by the clear extension of a single, radial process extending from cell bodies in the subgranular zone of the DG (examples are shown in Supplementary Videos 1–2). For the analysis of the modes of R and NR cell divisions, the definition of cell division modes is shown in Extended Data Fig. 3c. Only CERTAIN R and NR mother and daughter cells and certain transitions were considered. If a cell underwent cell death, the cell fate at the last time point before cell death was taken. Lineage tree visualization was performed using the igraph package (v.1.2.6) of R. The pheatmap package (v.1.0.12) of R was used for heat map visualization. Quantification graphs were visualized using GraphPad Prism (v.9.1.1).

### Analysis of neuronal migration and maturation

For the analysis of migratory behavior of newborn granule cells, continuous maximum projection files from individual imaging SPOTs were first aligned using the StackReg plugin function and then manually corrected for *x*/*y*-shifts in ImageJ. For the young group, neurons from seven SPOTs obtained from three animals (*n* = 40) were selected, and for the middle-aged group, neurons from seven SPOTs from five animals (*n* = 40) were selected. To trace individual newborn granule cells over time, cells were identified using the ROI codings of individual lineages, the Image5D files and corresponding lineage trees (as described above). Tracking of newborn granule cells over time was performed using the Manual tracking plugin function in ImageJ. For measurement of the direct displacement, a vector from the position of birth of a newborn granule cell (the last position of its mother cell) to its final destination at the last time point of imaging was drawn and measured. The soma size of an individual granule cell from this dataset was measured on collapsed maximum projections manually in ImageJ. The soma size of another granule cell in the same SPOT, which was considered a ‘leaky’ granule cell and was stable during the whole imaging session, was also measured and used as an ‘anchor cell’ for normalization. ‘Leaky’ granule cells were defined as small numbers of cells with granule cell morphology that were already TOM-positive at 2 dpi in both age groups (27.2 ± 9.8 cells in the young, *n* = 4; and 83.9 ± 14.1 cells in middle-aged mice, *n* = 4) and were negative for the expression of DCX, indicative of leaky expression of the TOM reporter in mature, preexisting granule cells (Extended Data Fig. 4c,d). The maturation processes of newborn granule cells over time were traced with the Simple Neurite Tracer plugin function in ImageJ. Tracing was performed on the neuronal morphology visible from the two-photon dataset of three SPOTs for the young group (*n* = 2) and eight SPOTs for the aged group (*n* = 5) based on the signal intensity of cells labeled as sufficient for the tracing of morphology. The following parameters were used: the length of total dendrites, the length of the longest branch, the length of the primary dendrite, and the number of dendritic branches.

### Tissue processing, immunostaining and confocal imaging

Mice were first anesthetized via i.p. injection of a lethal dose of pentobarbital and then transcardially perfused with cold saline, followed by 4% paraformaldehyde postfixed overnight at 4 °C. Brains were transferred to 30% sucrose solution for cryoprotection before being cut at a thickness of 40 μm (coronally) or 60 μm (horizontally) on a sliding microtome (Leica SM2010R). Every sixth coronal section (along the entire DG) and all horizontal sections were used for immunostaining. Sections were first washed in Tris-buffered saline (TBS) and blocked in staining buffer (3% donkey serum and 0.5% Triton X-100 in TBS) for 1 h at room temperature. Then, sections were incubated with primary antibodies against GFP (1:500, goat; Rockland), Ki67 (1:500, rat; Thermo Fisher Scientific), DCX (1:500, guinea pig; Millipore), SOX2 (1:500, rabbit; Millipore), SOX2 (1:200, rat; Thermo Fisher Scientific), GFAP (1:500, chicken; Aves), S100B (1:500, rabbit; Abcam) and tdTomato (1:500, goat; Sicgen) for two nights in staining buffer at 4 °C. After washing in TBS, sections were incubated with secondary antibodies against the respective species (Alexa Fluro 488, Cy3 and Cy5, 1:250) and DAPI (1 μg ml^−1^, Thermo) in staining buffer for 2 h at room temperature. After washing, sections were mounted with Immun-Mount (Thermo) and stored at 4 °C until imaging. Images were taken using confocal laser scanning microscopes (Zeiss LSM800 with ZEN 2.3 software). Antibodies are specified with details in the reporting summary.

### Statistics and reproducibility

All results in graphs are presented as mean ± s.e.m. unless specified otherwise. Statistical analyses were performed in GraphPad Prism (v.9.1.1) or R (v.3.6.3). For pairwise comparisons between two groups, two-tailed unpaired *t* test with Welsh’s correction or two-tailed Mann–Whitney test was performed. For multiple time point comparisons between two groups, two-way analysis of variance (ANOVA) was performed. For comparisons of global trends between two groups, Kolmogorov–Smirnov test was performed. For comparison of cluster composition, chi-square or Fisher’s exact test were performed. For statistical significance was determined based on *P*-value (not significant, *P* > 0.05; *P < 0.05; **P < 0.01; ***P< 0.001; ****P < 0.0001). Particular tests and statistical significance for individual comparisons shown in figures are detailed in the Supplementary Table 1.

No statistical methods were used to predetermine sample sizes; these were derived from previous publications^4,5^. Only CERTAIN cell phenotypes and transitions were included in subsequent analyses as detailed above (Methods). The experiments were not randomized owing to the two different age cohorts. The investigators were not blinded to allocation during intravital imaging acquisition but were blinded during the outcome assessment and analyses.

### Reporting summary

Further information on research design is available in the Nature Portfolio Reporting Summary linked to this article.

## Supplementary information

Supplementary Information Supplementary Fig. 1.

Reporting Summary

Supplementary Video 1 Representative video showing the identification of R and NR cells in the middle-aged DG.

Supplementary Video 2 Representative video showing the activation and proliferation of an aged NSC, and the migration and death of its progeny.

Supplementary Video 3 An example video depicting the tracking of the migration trajectory of newborn granule cells.

Supplementary Video 4 An example video showing the identification of neuronal progeny in the middle-aged DG.

Supplementary Table 1 Statistical details for main figures and extended data figures.

Supplementary Table 2 Number of z-stacks of collapsed two-photon images for main figures and extended data figures.

## Data Availability

Data generated and analyzed during this study are included in the published article (and its supplementary information and source files) or available from the corresponding author on reasonable request. Data for lineage analysis are available at https://github.com/JessbergerLab/AgingNeurogenesis_Imaging.

## Source data

Source Data Fig. 1 Numerical source data.
Source Data Fig. 2 Numerical source data.
Source Data Fig. 3 Numerical source data.
Source Data Fig. 4 Numerical source data.
Source Data Extended Data Fig. 1 Numerical source data.
Source Data Extended Data Fig. 3 Numerical source data.
Source Data Extended Data Fig. 4 Numerical source data.

## Code availability

Custom code (HippoLinTools) used to analyze the cellular behavior is available at https://github.com/JessbergerLab/AgingNeurogenesis_Imaging. Custom code was developed by M. Betizeau.
