# Supplementary material for: Chronic in vivo imaging defines age-dependent alterations of neurogenesis in the mouse hippocampus
Source: Nat Aging. 2023 Feb 20;3(4):380–90. doi: 10.1038/s43587-023-00370-9 (PMC10154232; doi:10.1038/s43587-023-00370-9)

nature aging

Letter

https://doi.org/10.1038/s43587-023-00370-9

Chronic in vivo imaging defines age-dependent alterations of neurogenesis in the mouse hippocampus

In the format provided by the authors and unedited

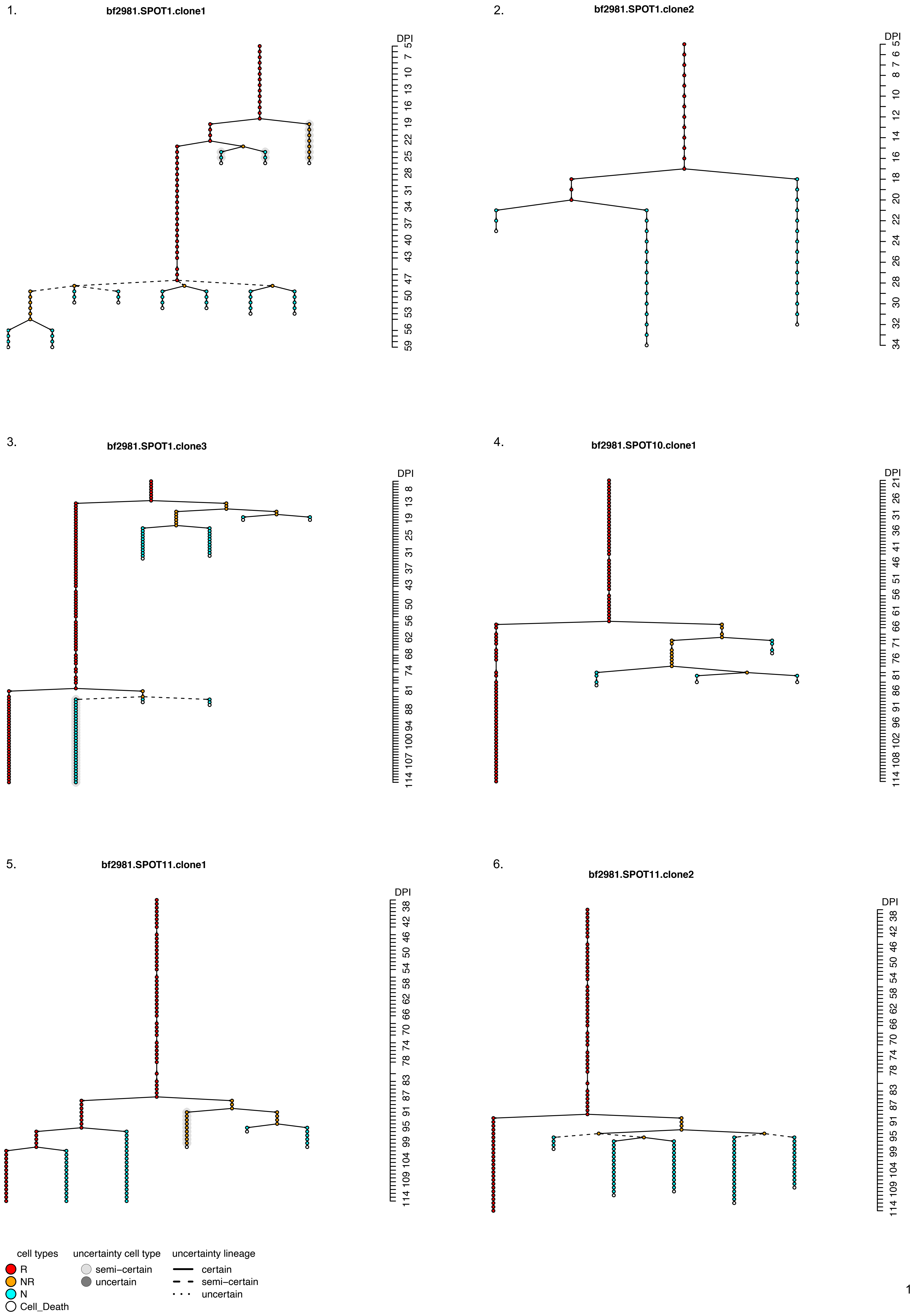
1.
bf2981.SPOT1.clone1
2.
bf2981.SPOT1.clone2
3.
bf2981.SPOT1.clone3
4.
bf2981.SPOT10.clone1
5.
bf2981.SPOT11.clone1
6.
bf2981.SPOT11.clone2
DPI
cell types
R
NR
N
Cell_Death
uncertainty cell type
semi−certain
uncertain
uncertainty lineage
certain
semi−certain
uncertain

7. **bf2981.SPOT3.clone1**

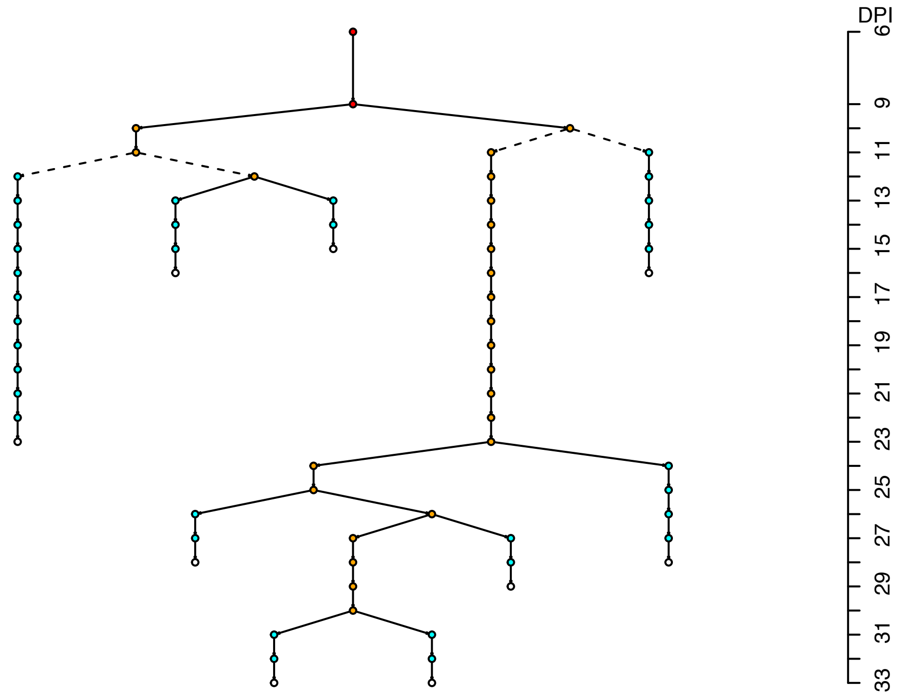

8. **bf2981.SPOT3.clone2**

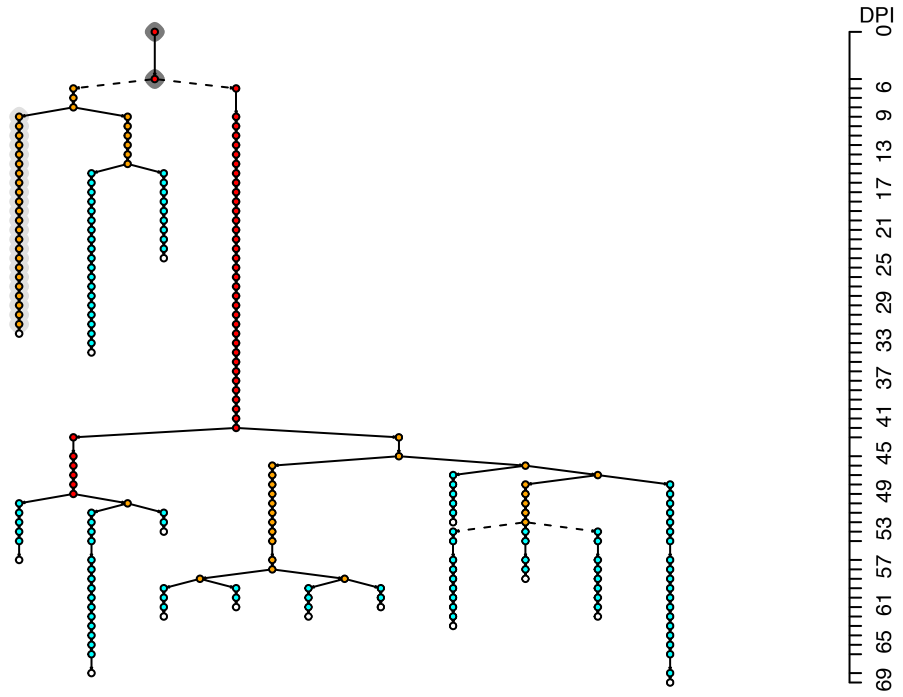

9. **bf2981.SPOT5.clone1**

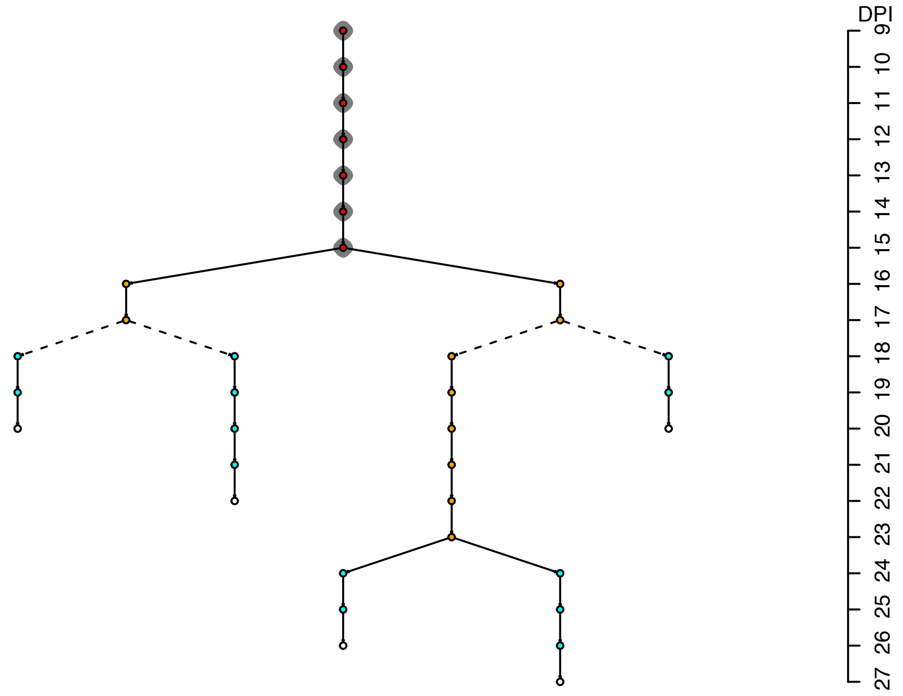

10. **bf2981.SPOT5.clone2**

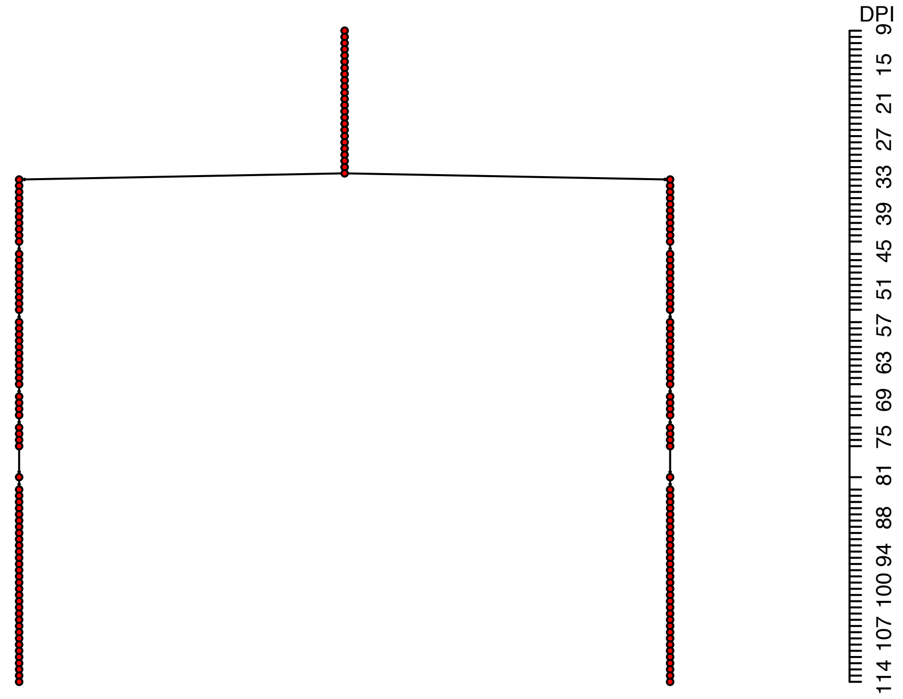

11. **bf2981.SPOT5.clone3**

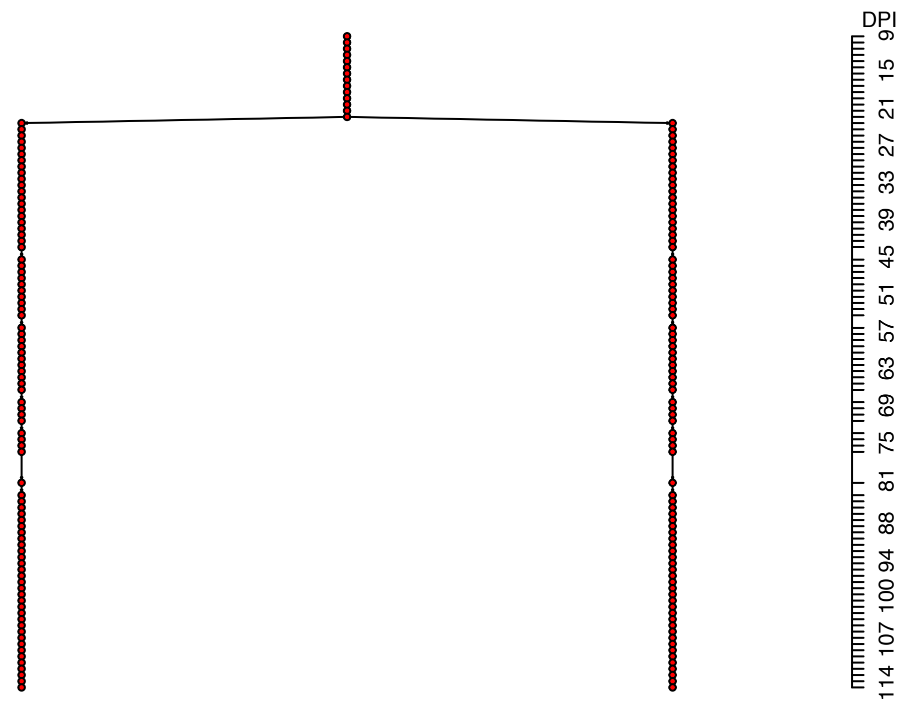

12. **bf2981.SPOT6.clone1**

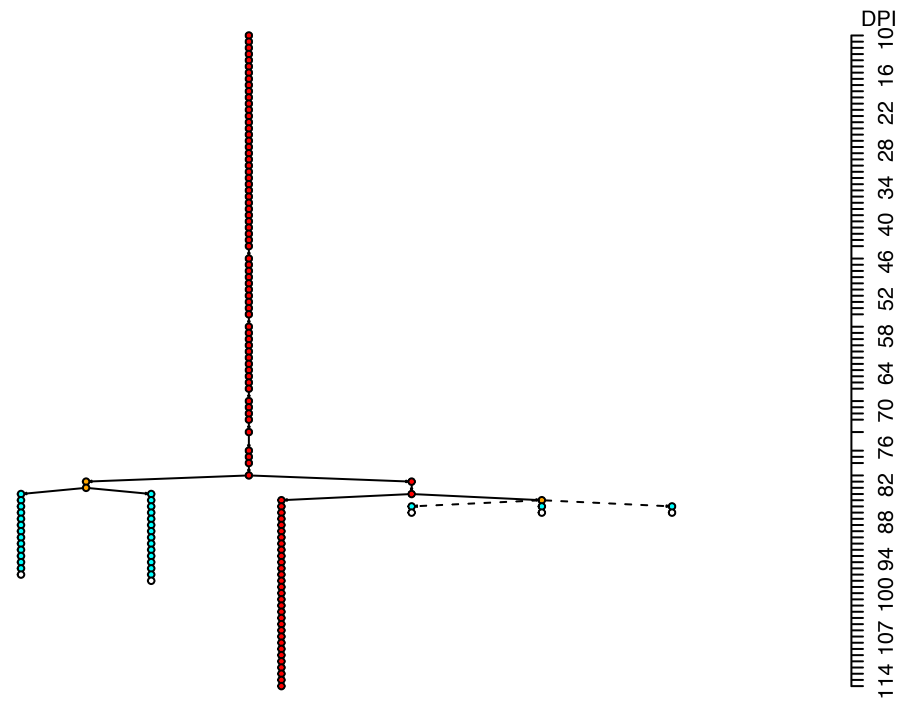

cell types	uncertainty cell type	uncertainty lineage
R	semi−certain	certain
NR	uncertain	semi−certain
N		uncertain
Cell_Death		

13. **bf2981.SPOT6.clone2**

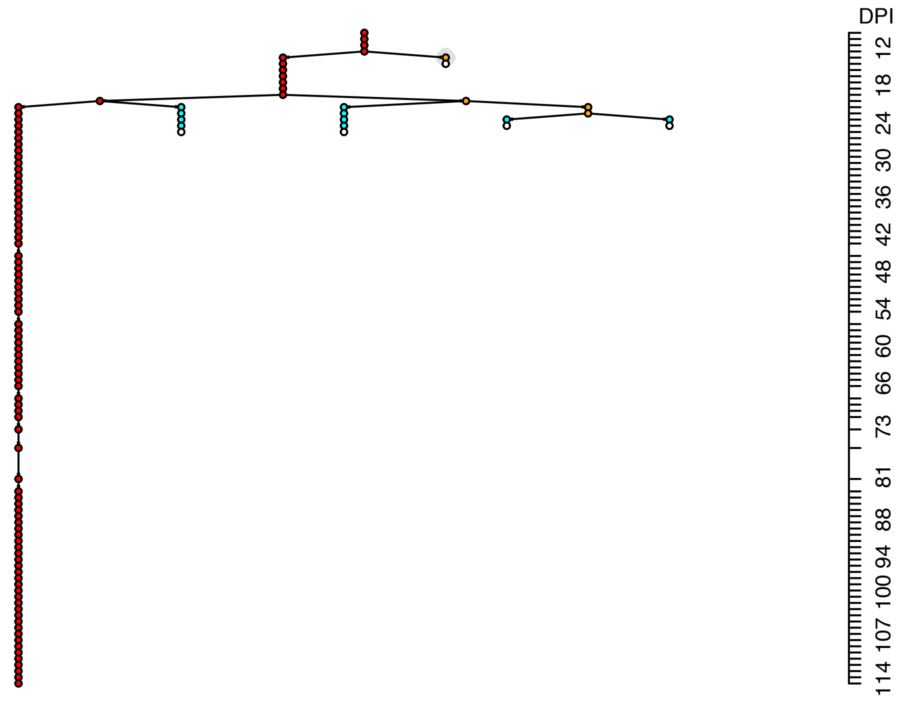

14. **bf2981.SPOT6.clone3**

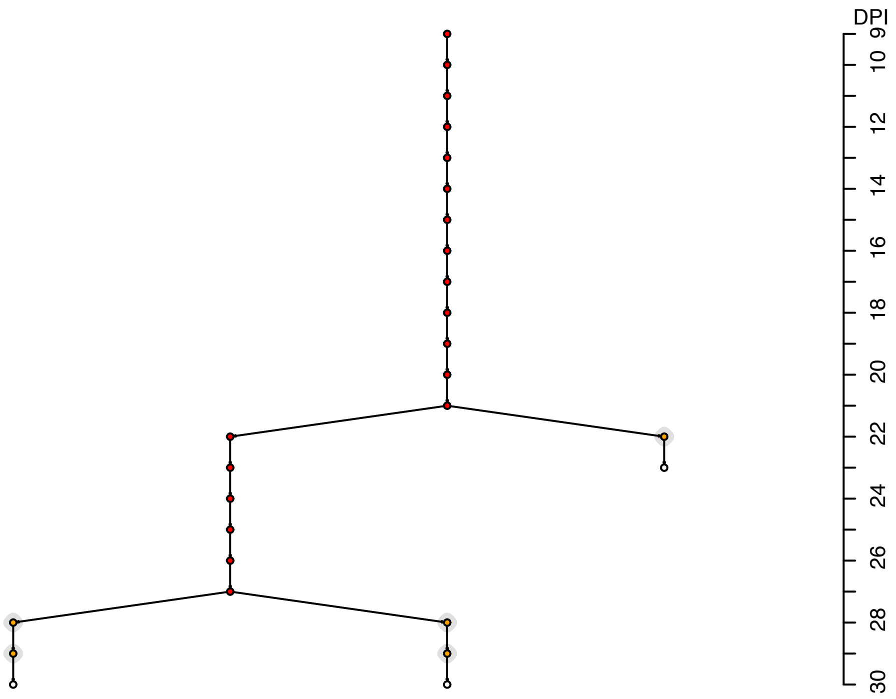

15. **bf2983.SPOT1.clone1**

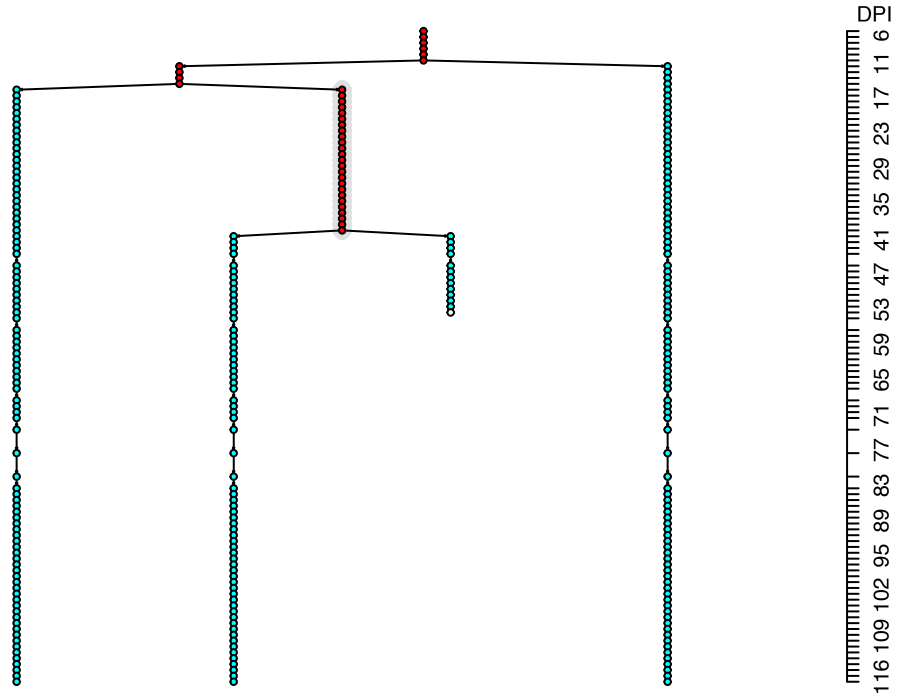

16. **bf2983.SPOT1.clone2**

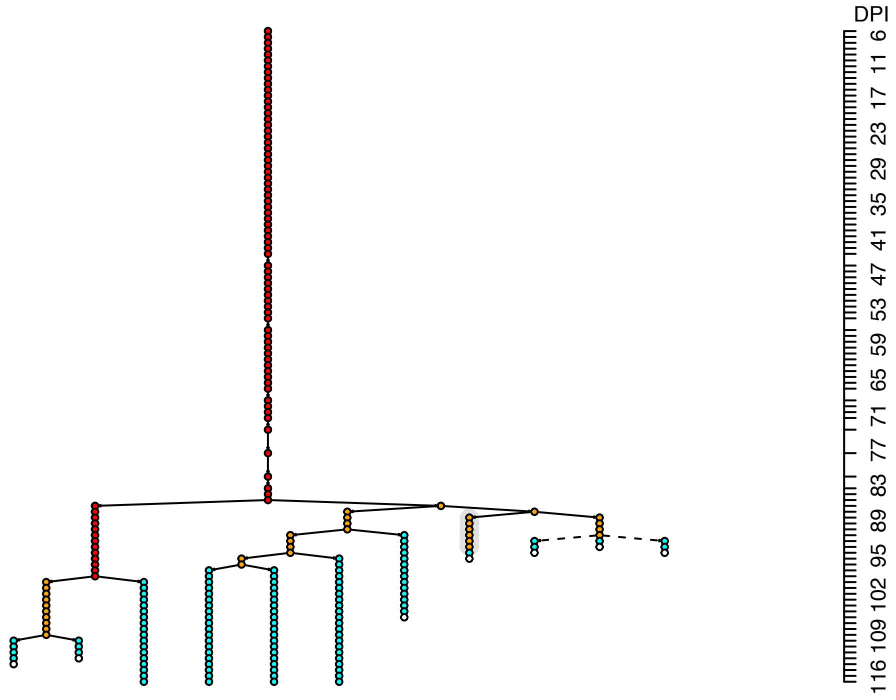

17. **bf2983.SPOT1.clone3**

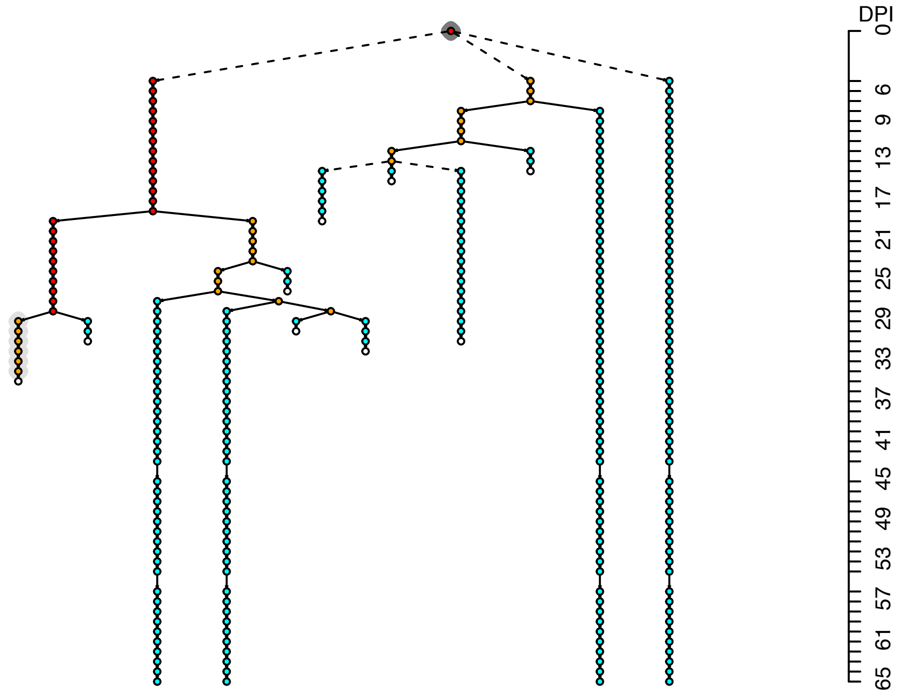

18. **bf2983.SPOT10.clone1**

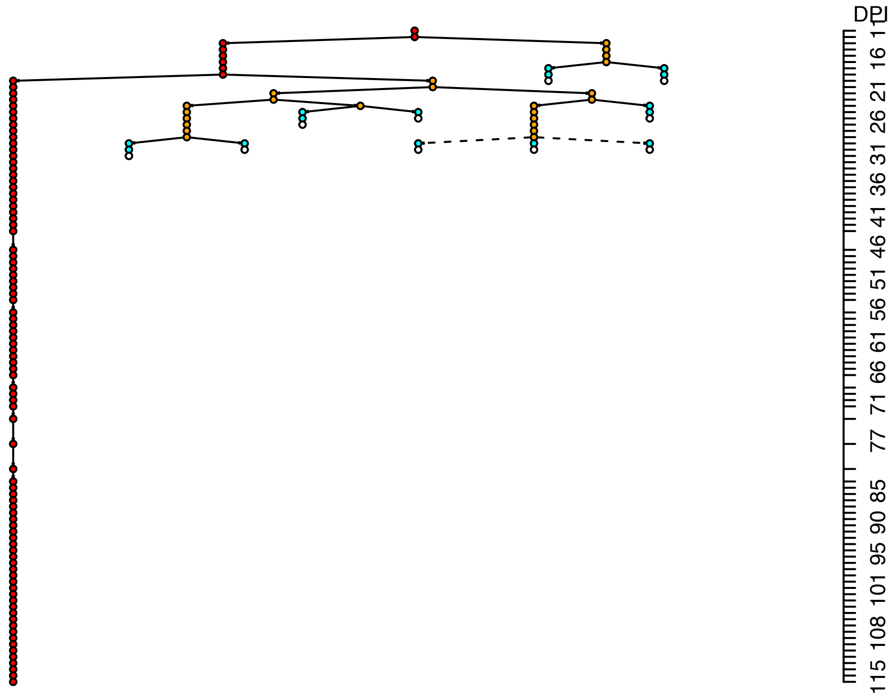

cell types: R, NR, N, Cell_Death
uncertainty cell type: semi–certain, uncertain
uncertainty lineage: certain, semi–certain, uncertain

19. **bf2983.SPOT10.clone2**

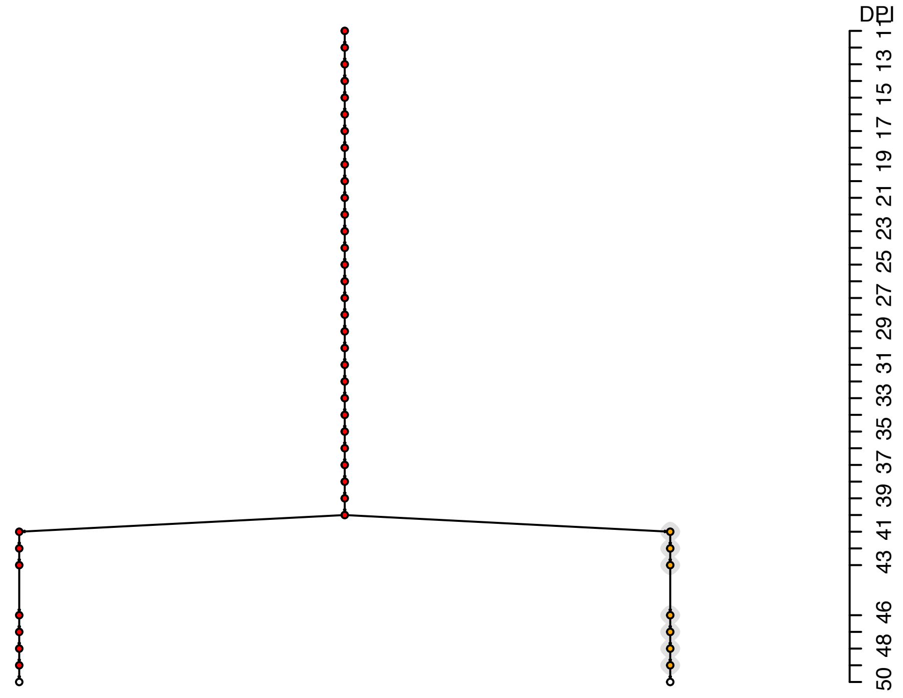

20. **bf2983.SPOT2.clone1**

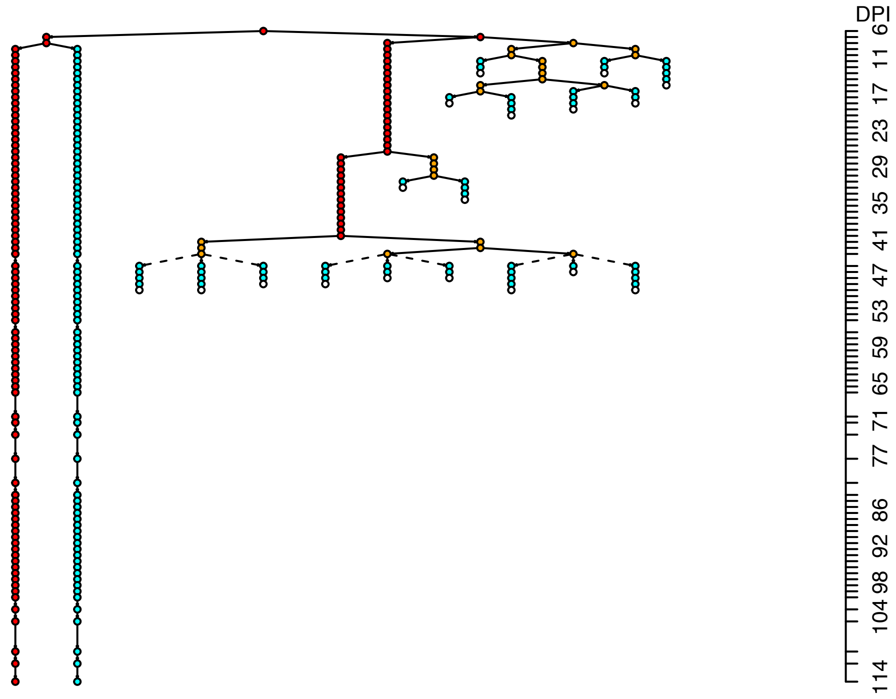

21. **bf2983.SPOT2.clone2**

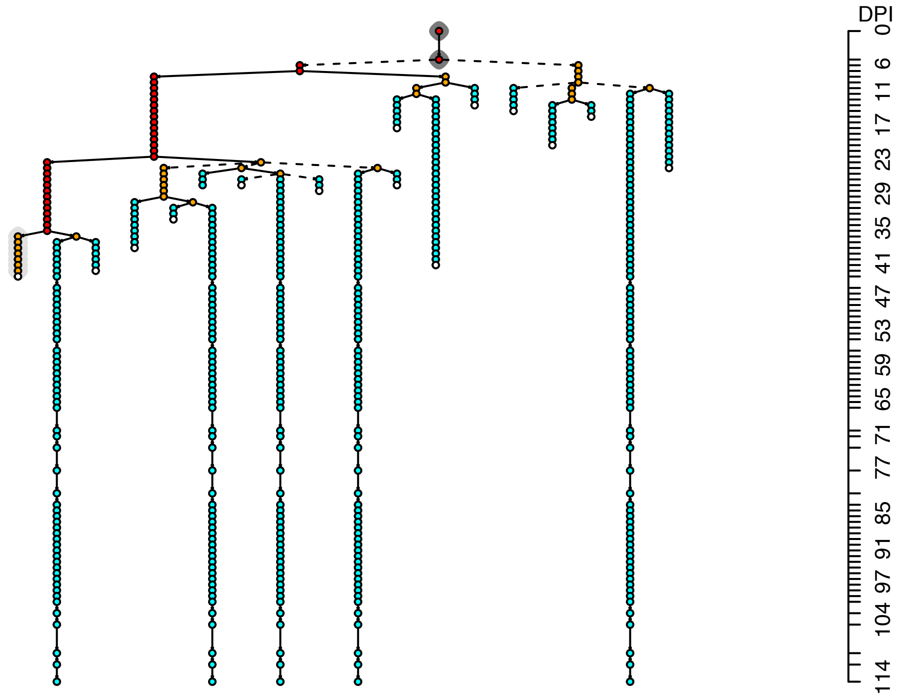

22. **bf2983.SPOT3.clone1**

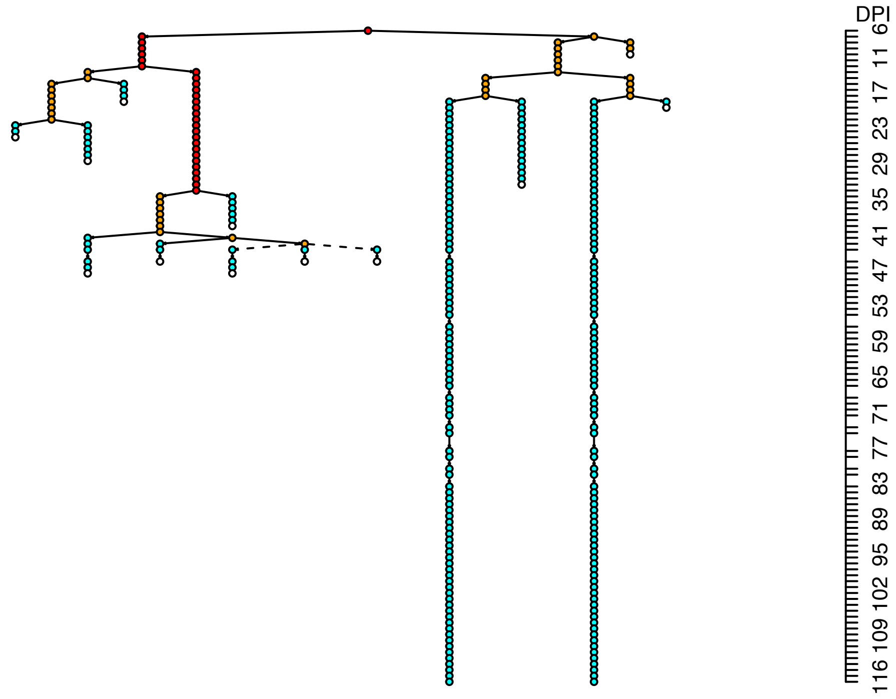

23. **bf2983.SPOT5.clone1**

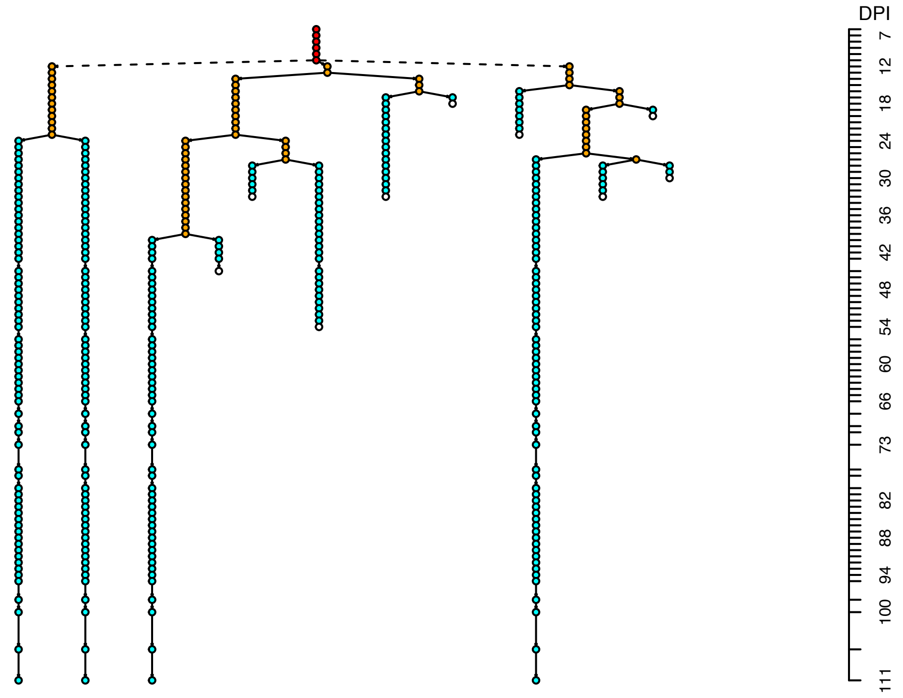

24. **bf2983.SPOT5.clone2**

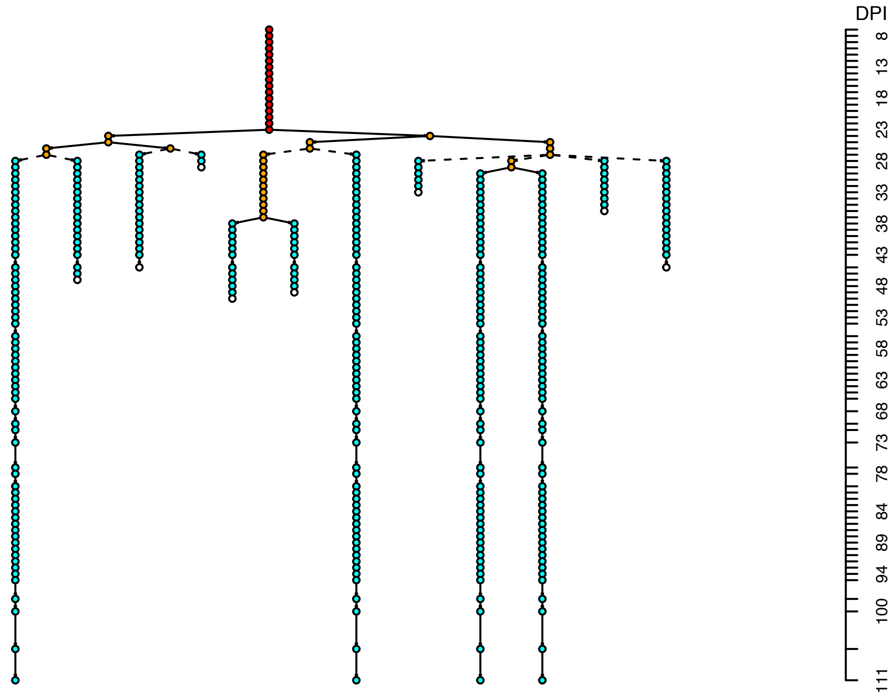

cell types: R, NR, N, Cell_Death

uncertainty cell type: semi−certain, uncertain

uncertainty lineage: certain, semi−certain, uncertain

25. **bf2983.SPOT7.clone1**

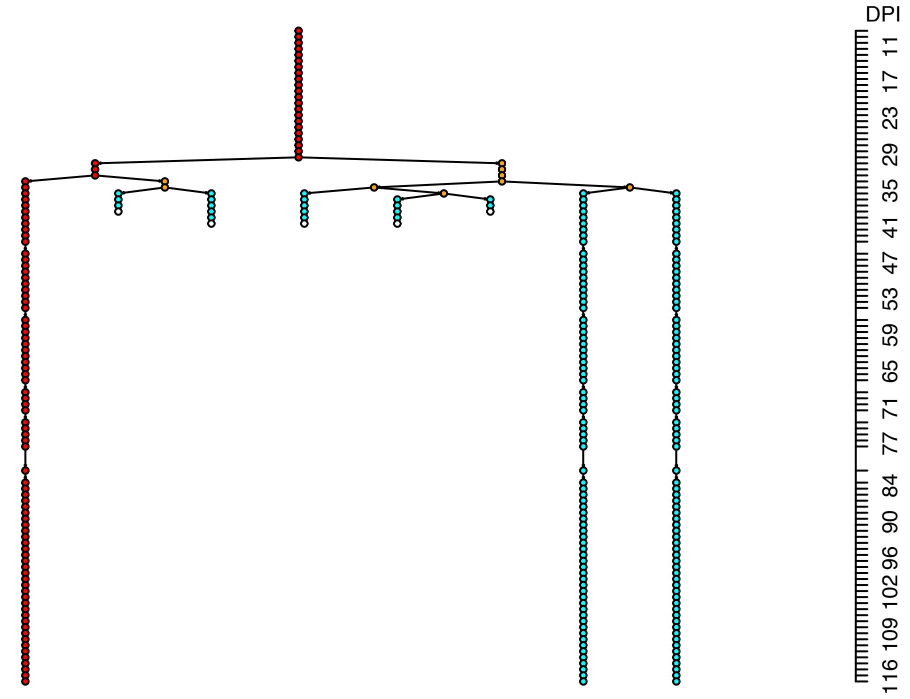

26. **bf2983.SPOT7.clone2**

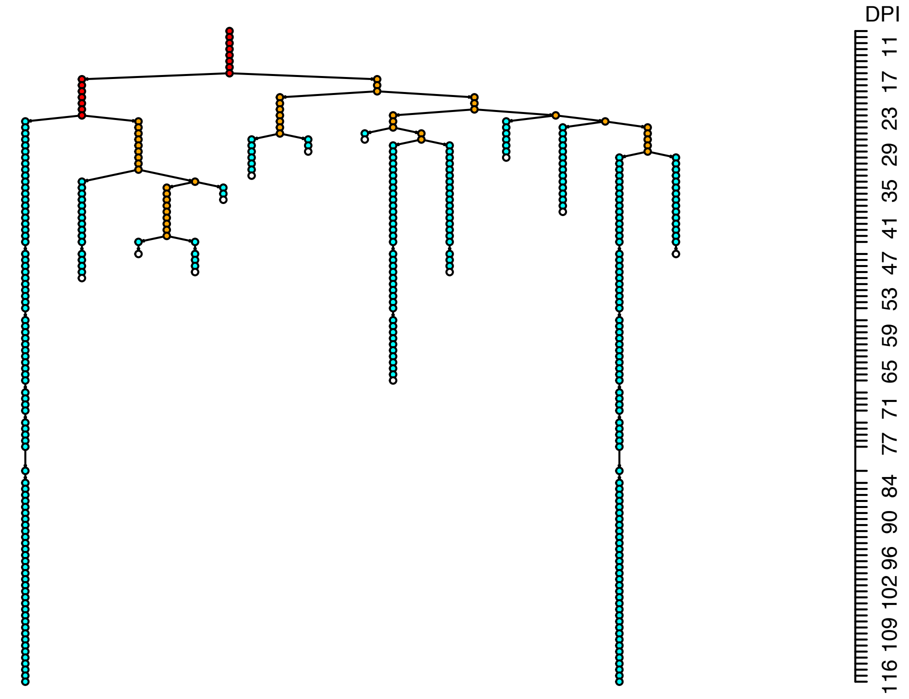

27. **bf2983.SPOT7.clone3**

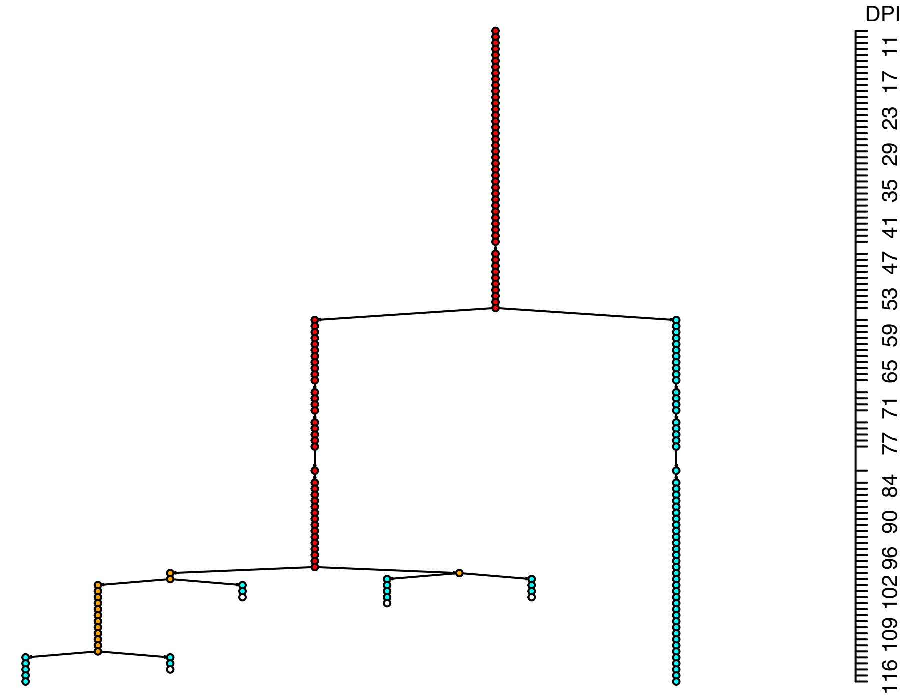

28. **bf2983.SPOT7.clone5**

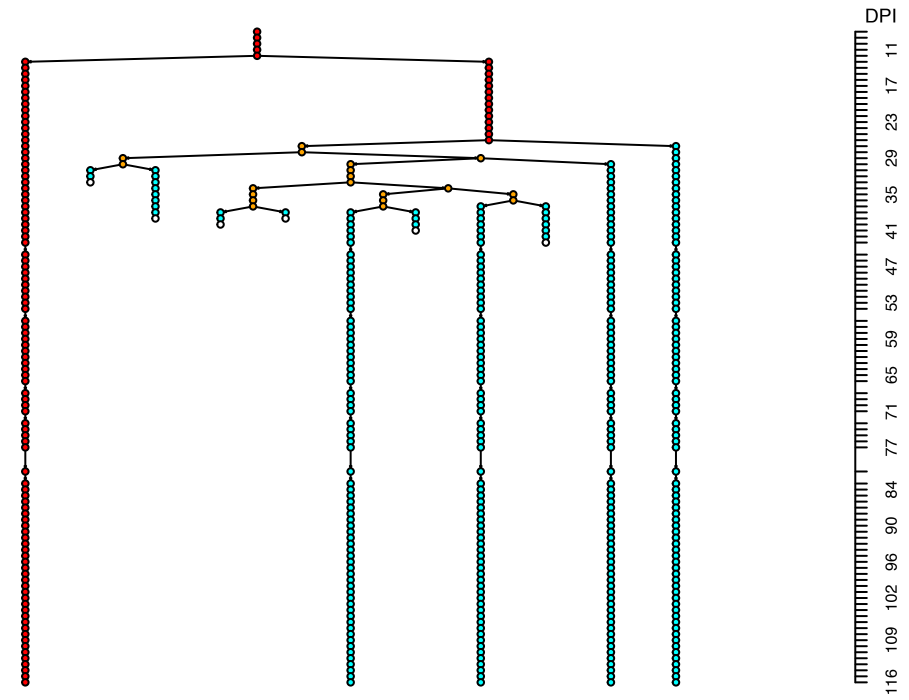

29. **bf2983.SPOT7.clone6**

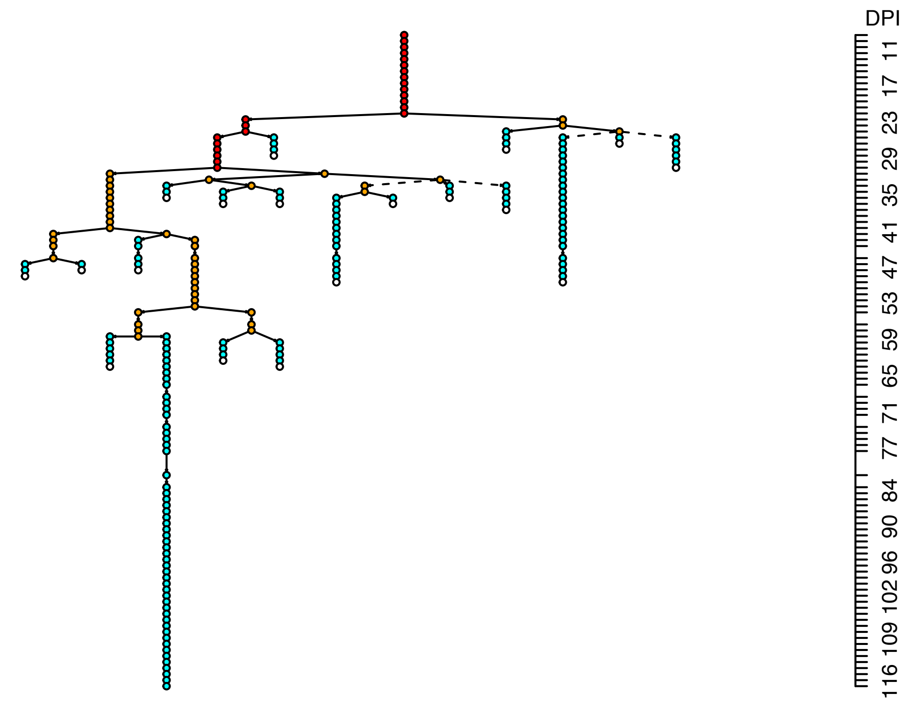

30. **bf2985.SPOT14.clone1**

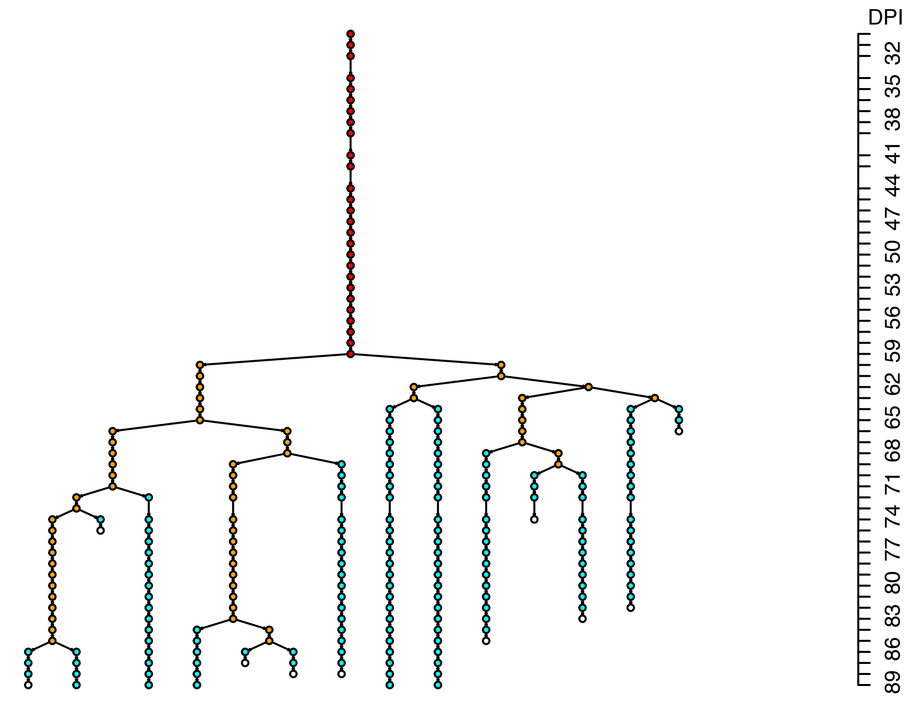

cell types: R, NR, N, Cell_Death

uncertainty cell type: semi−certain, uncertain

uncertainty lineage: certain, semi−certain, uncertain

31. **bf2985.SPOT2.clone1**

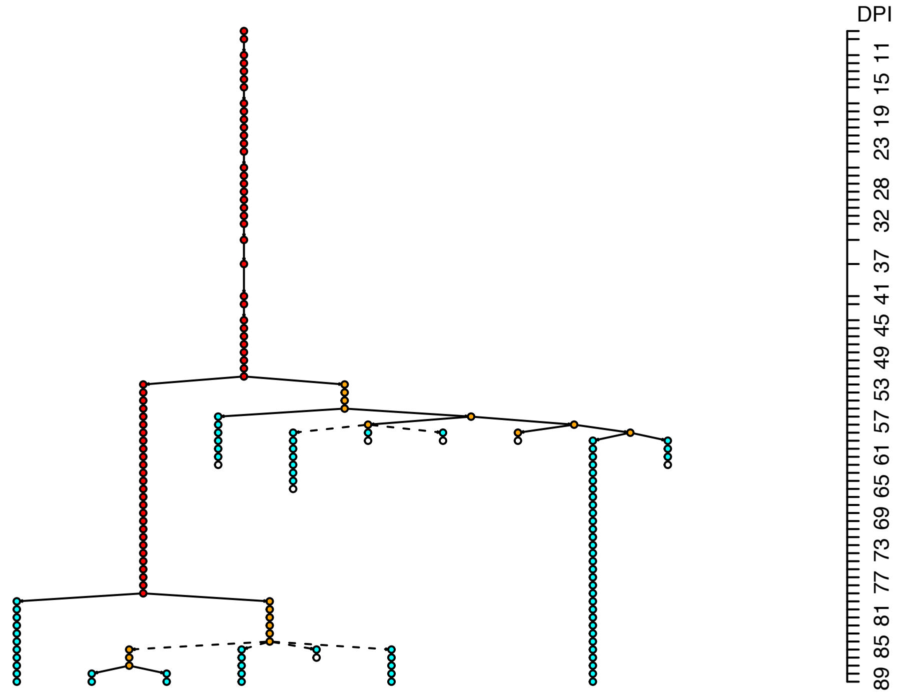

32. **bf2985.SPOT4.clone1**

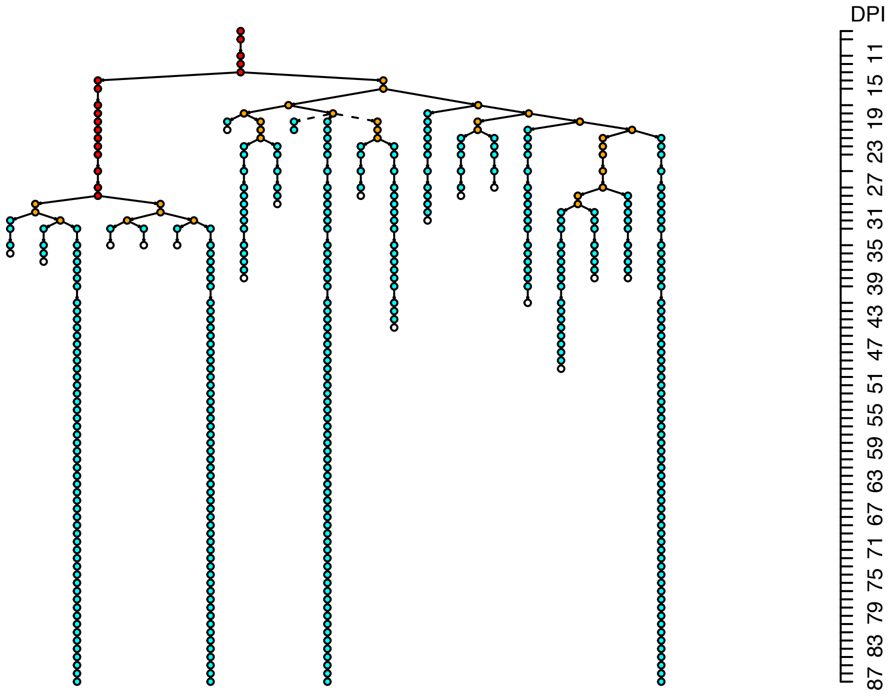

33. **bf2985.SPOT4.clone2**

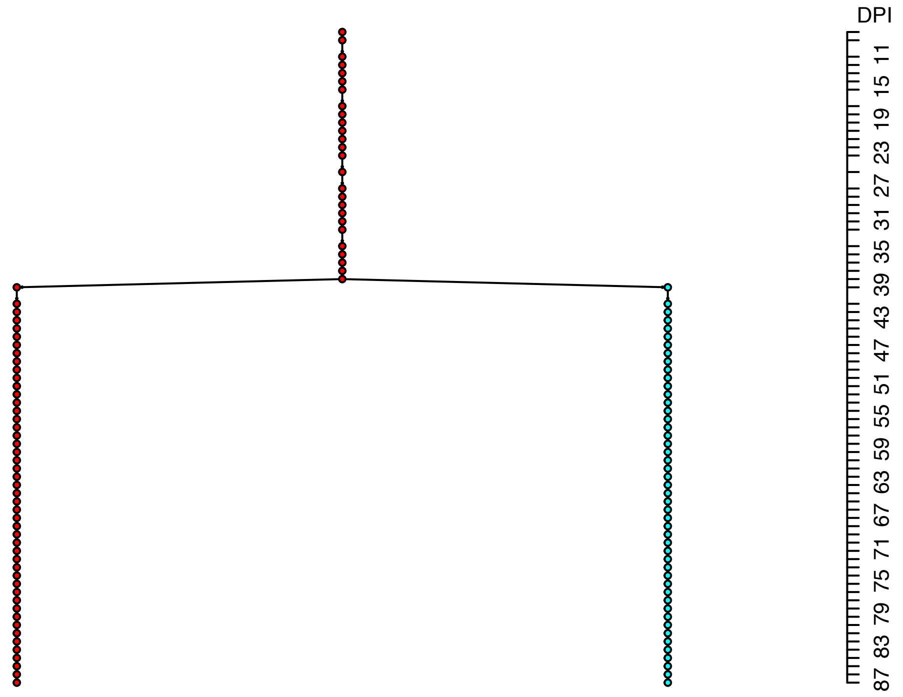

34. **bf2985.SPOT4.clone3**

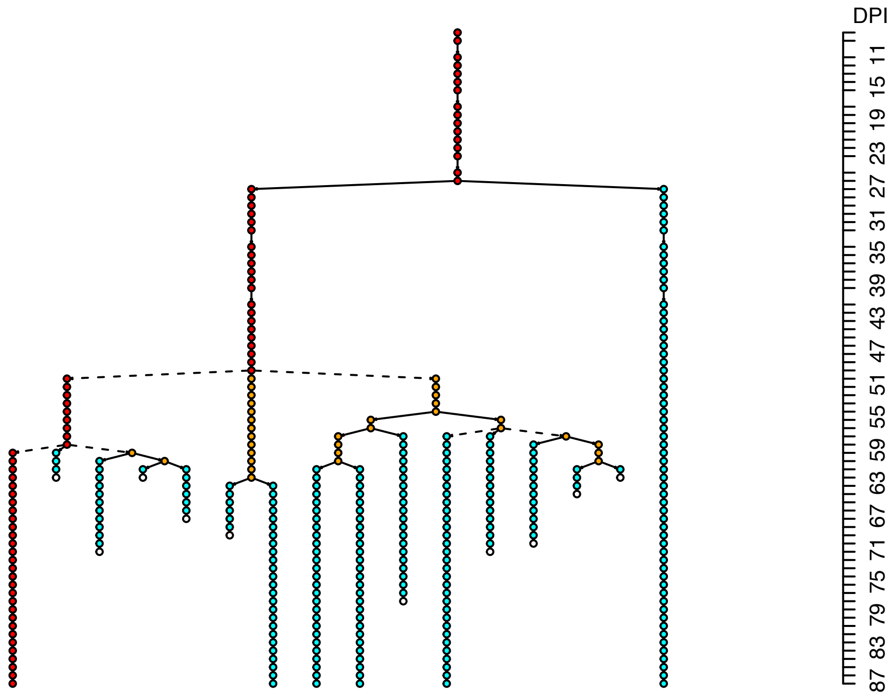

35. **bf2985.SPOT4.clone5**

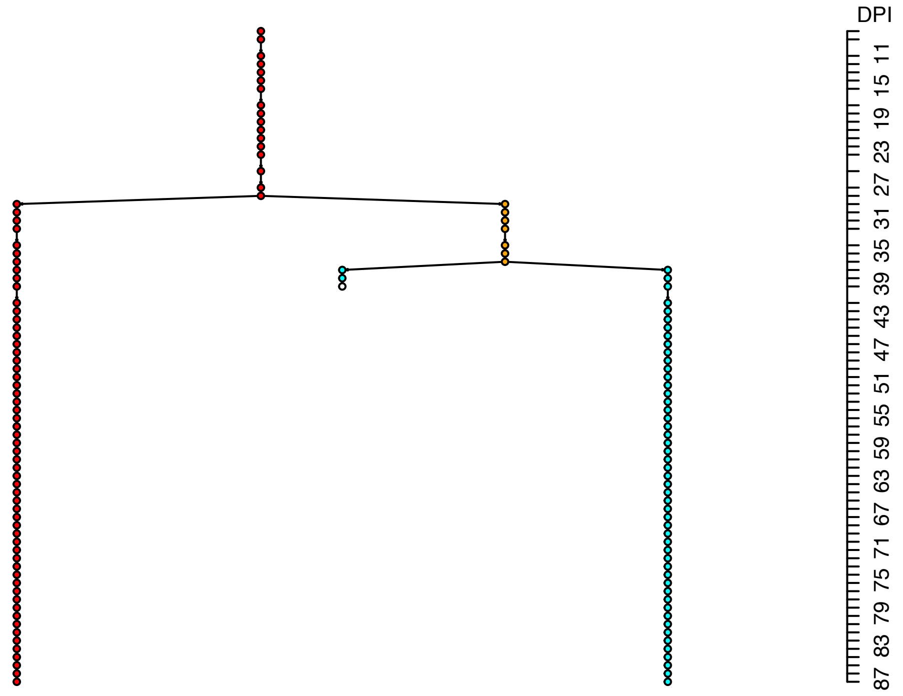

36. **bf2985.SPOT4.clone7**

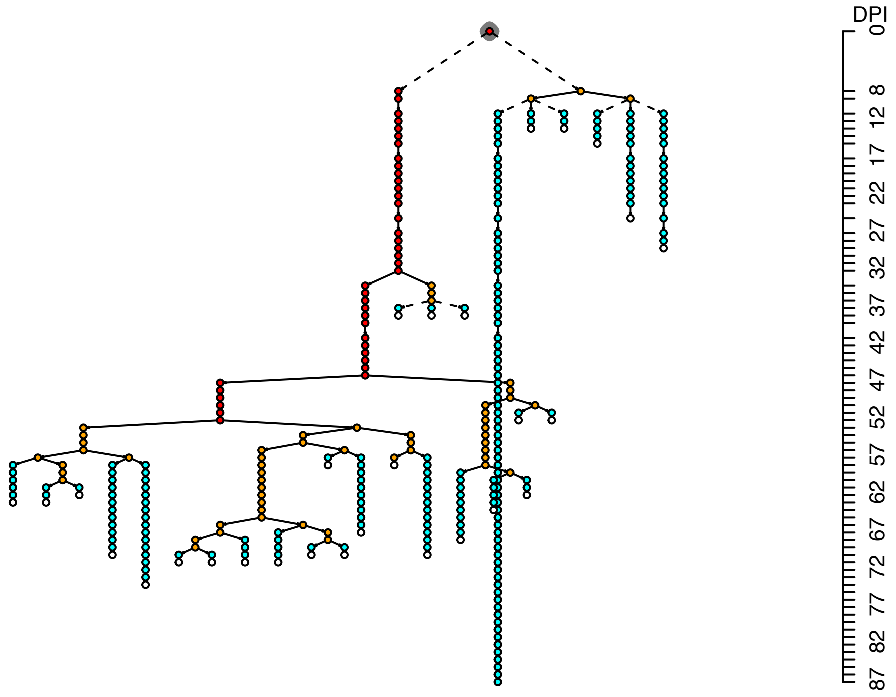

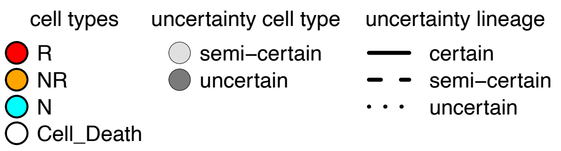

37. **bf3419.SPOT12.clone1**

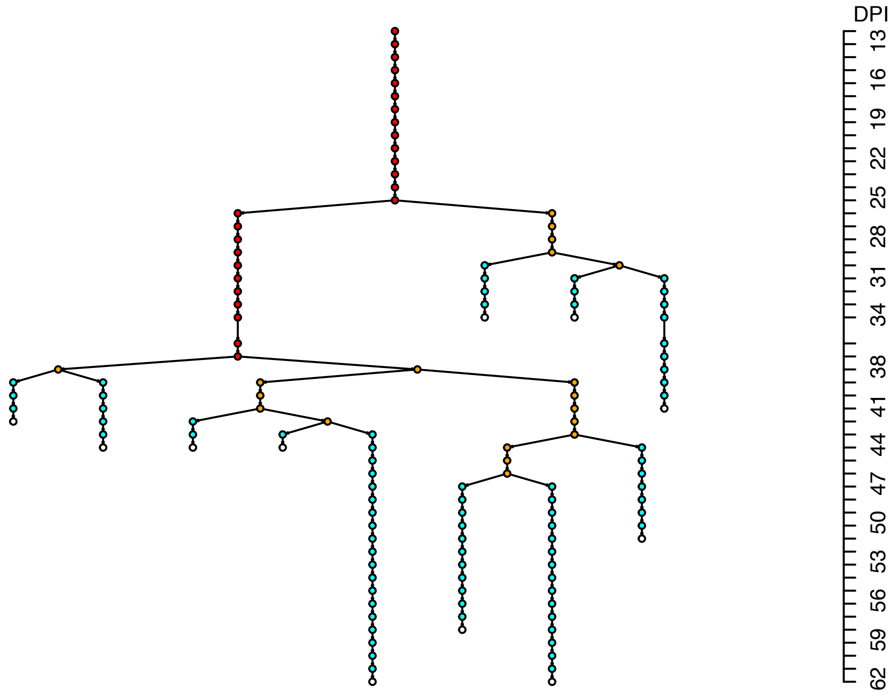

38. **bf3419.SPOT12.clone2**

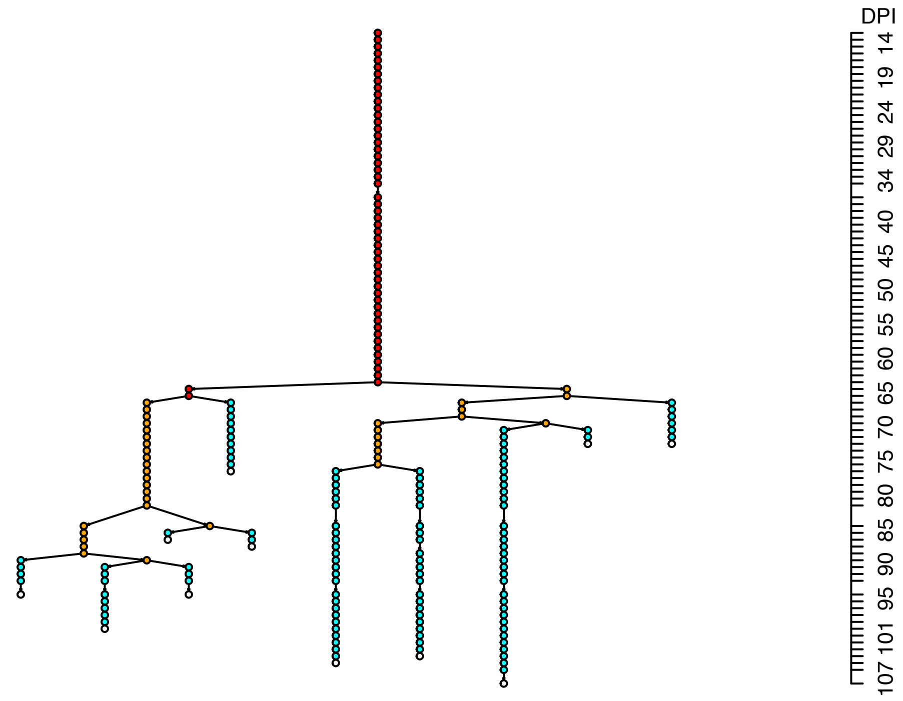

39. **bf3419.SPOT12.clone3**

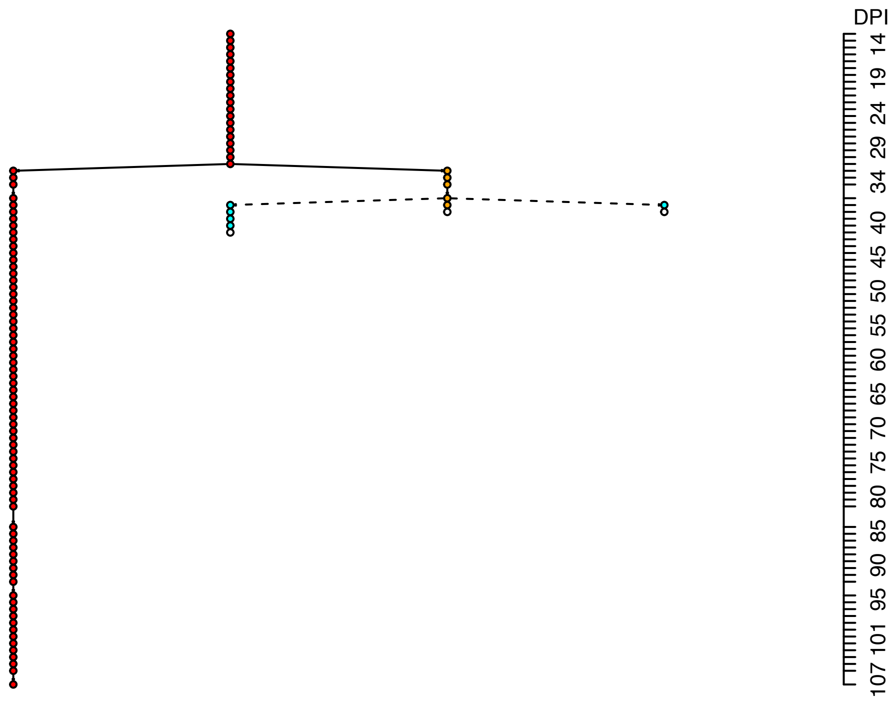

40. **bf3419.SPOT14.clone1**

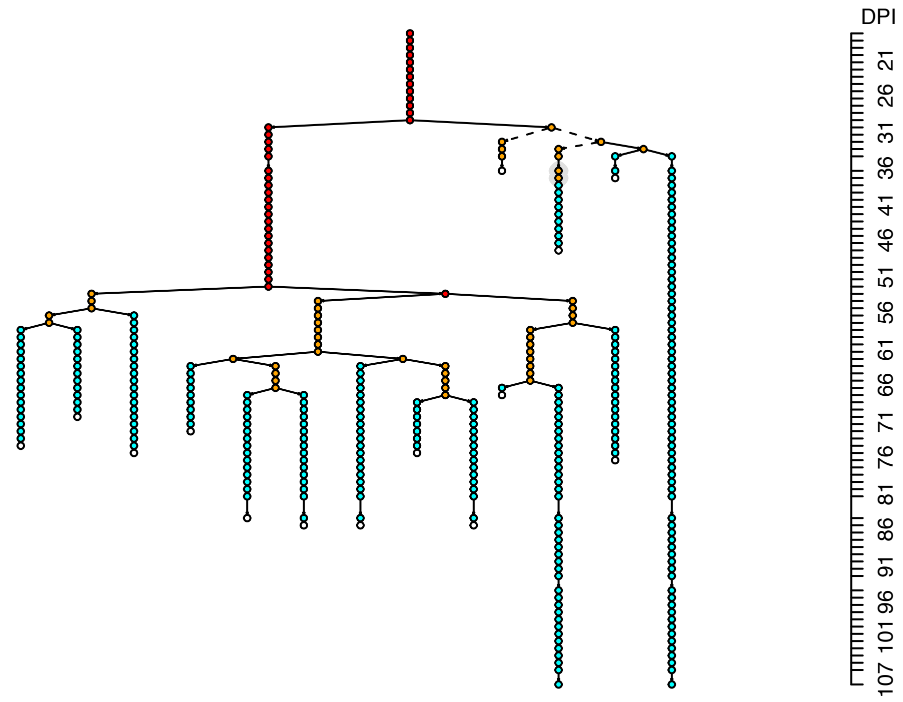

41. **bf3419.SPOT6.clone1**

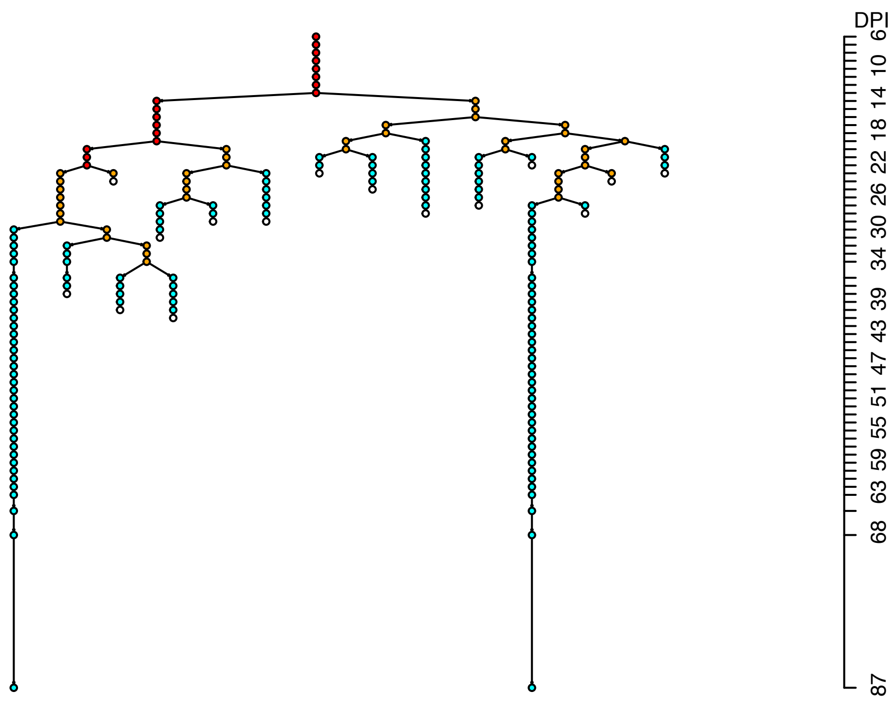

42. **bf3419.SPOT6.clone2**

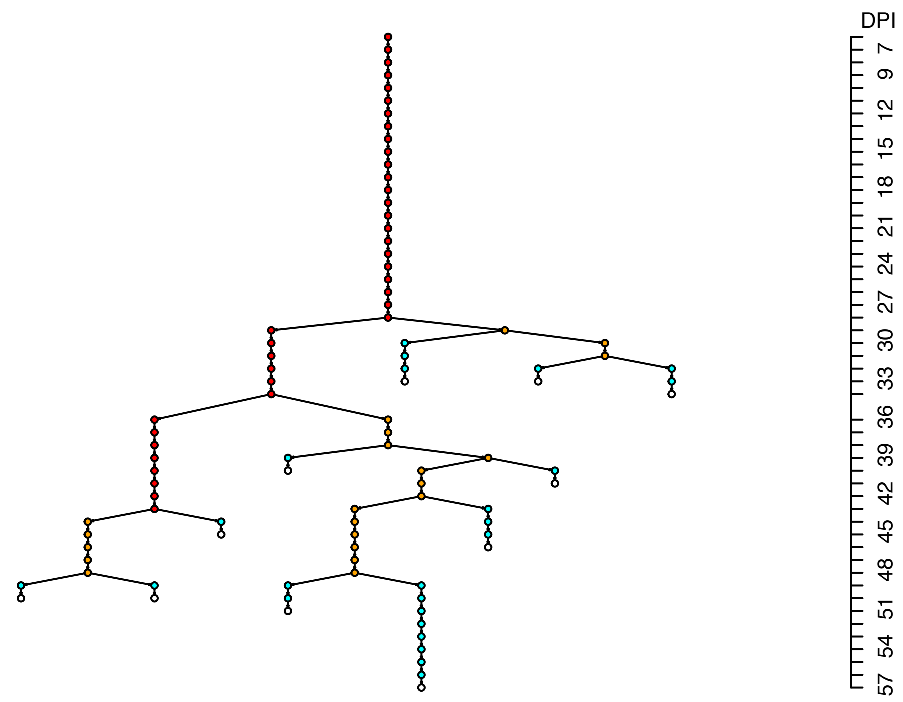

cell types	uncertainty cell type	uncertainty lineage
R	semi–certain	certain
NR	uncertain	semi–certain
N		uncertain
Cell_Death		

43.

bf3419.SPOT7.clone1

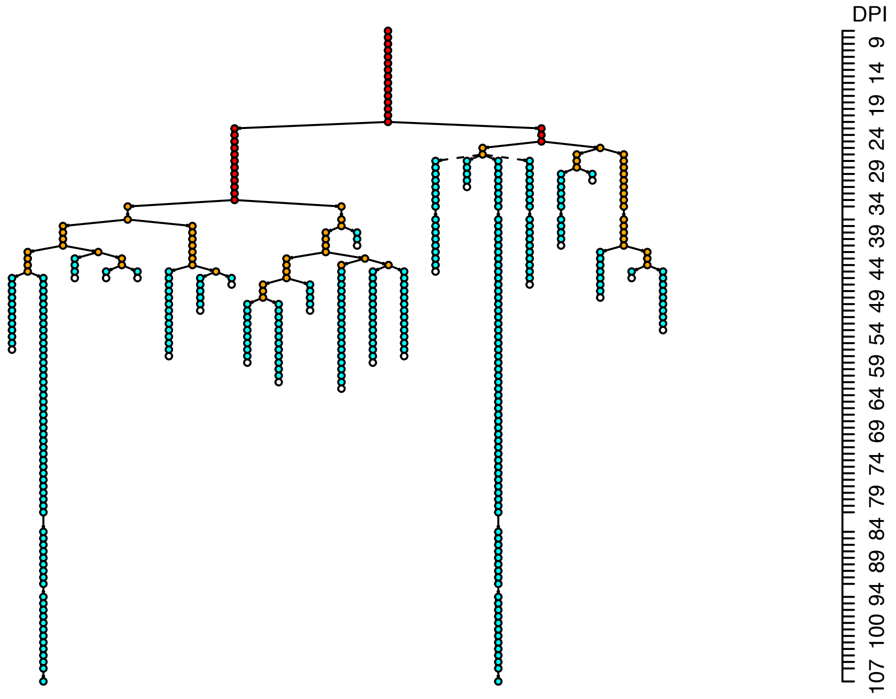

44.

bf4845.SPOT3.clone1

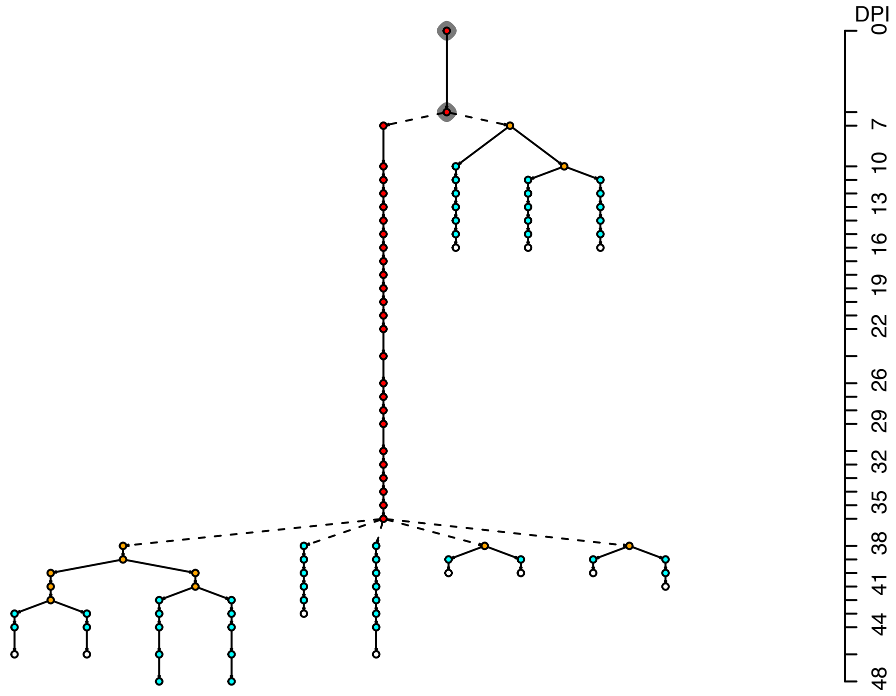

45.

bf4845.SPOT4.clone1

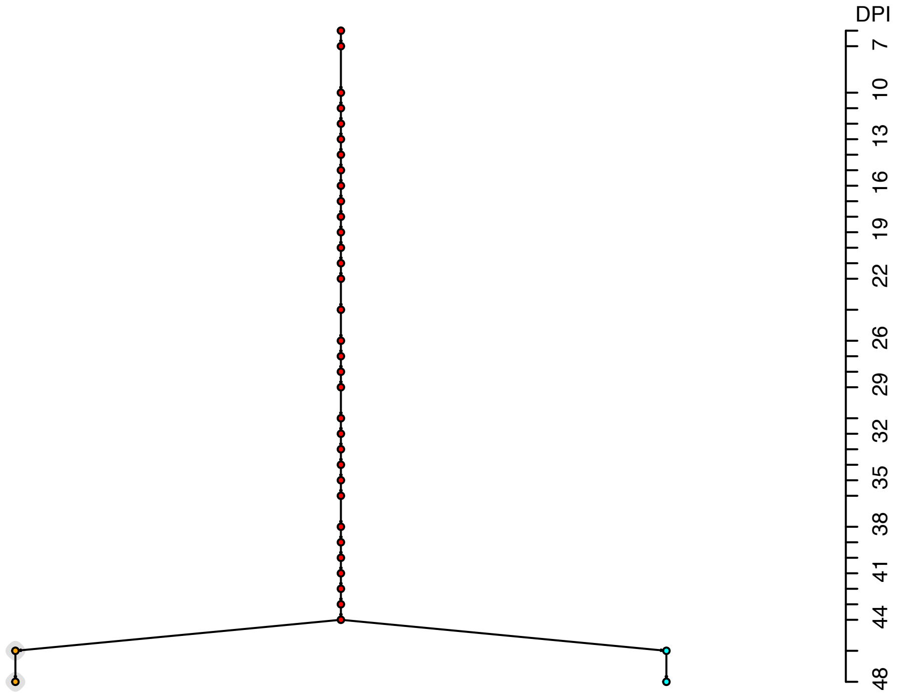

46.

bf4845.SPOT4.clone2

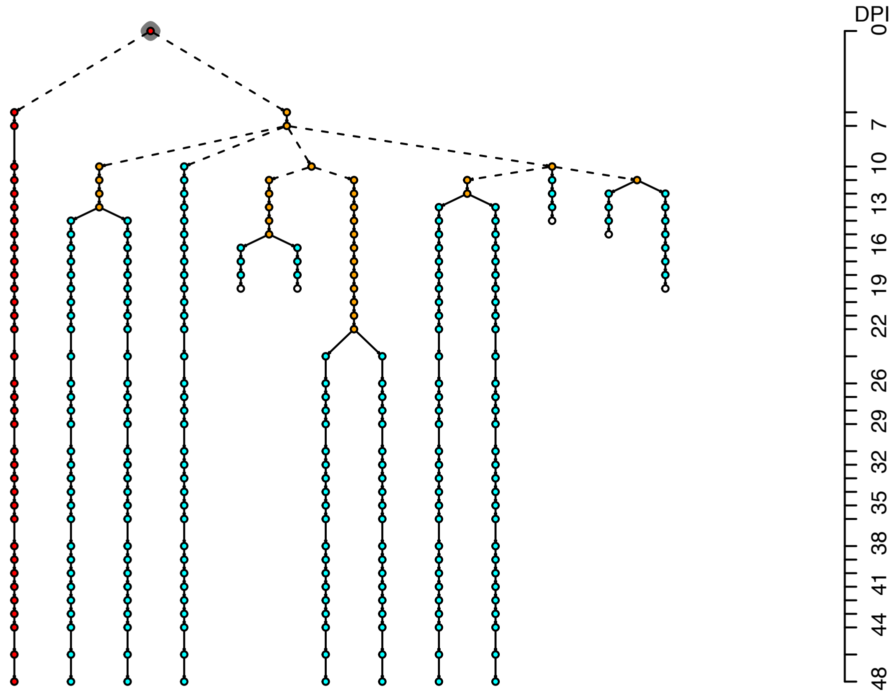

47.

bf4845.SPOT4.clone3

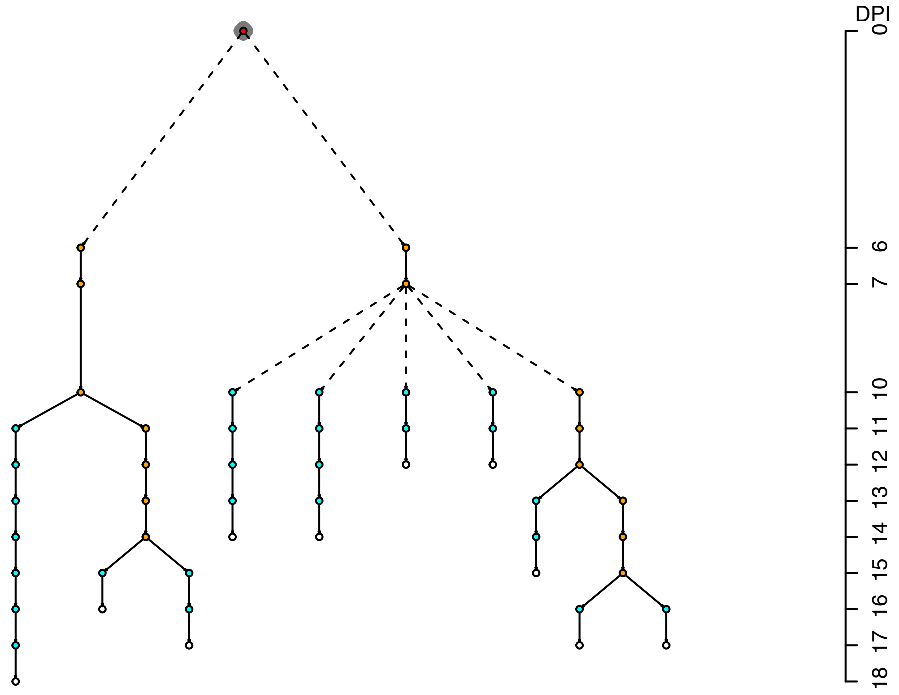

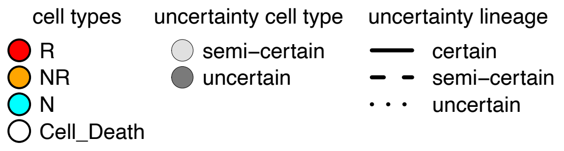

Supplement: Supplementary file 1 — Supplementary Fig. 1. [file 43587_2023_370_MOESM1_ESM.pdf]
