# Supplementary material for: Chronic in vivo imaging defines age-dependent alterations of neurogenesis in the mouse hippocampus
Source: Nat Aging. 2023 Feb 20;3(4):380–90. doi: 10.1038/s43587-023-00370-9 (PMC10154232; doi:10.1038/s43587-023-00370-9)
Supplement: Supplementary file 2 — Reporting Summary [file 43587_2023_370_MOESM2_ESM.pdf]

nature portfolio

Corresponding author(s): Sebastian Jessberger

Last updated by author(s): 2022/03/11

Reporting Summary

Nature Portfolio wishes to improve the reproducibility of the work that we publish. This form provides structure for consistency and transparency in reporting. For further information on Nature Portfolio policies, see our Editorial Policies and the Editorial Policy Checklist.

Statistics

For all statistical analyses, confirm that the following items are present in the figure legend, table legend, main text, or Methods section.

n/a	Confirmed	
☐	☒	The exact sample size (*n*) for each experimental group/condition, given as a discrete number and unit of measurement
☐	☒	A statement on whether measurements were taken from distinct samples or whether the same sample was measured repeatedly
☐	☒	The statistical test(s) used AND whether they are one- or two-sided<br>*Only common tests should be described solely by name; describe more complex techniques in the Methods section.*
☐	☒	A description of all covariates tested
☐	☒	A description of any assumptions or corrections, such as tests of normality and adjustment for multiple comparisons
☐	☒	A full description of the statistical parameters including central tendency (e.g. means) or other basic estimates (e.g. regression coefficient) AND variation (e.g. standard deviation) or associated estimates of uncertainty (e.g. confidence intervals)
☐	☒	For null hypothesis testing, the test statistic (e.g. *F*, *t*, *r*) with confidence intervals, effect sizes, degrees of freedom and *P* value noted<br>*Give P values as exact values whenever suitable.*
☒	☐	For Bayesian analysis, information on the choice of priors and Markov chain Monte Carlo settings
☒	☐	For hierarchical and complex designs, identification of the appropriate level for tests and full reporting of outcomes
☒	☐	Estimates of effect sizes (e.g. Cohen's *d*, Pearson's *r*), indicating how they were calculated

Our web collection on statistics for biologists contains articles on many of the points above.

Software and code

Policy information about availability of computer code

Data collection	ZEN Version 2.3 Carl Zeiss AG http://www.zeiss.com/microscopy/en_us/products/microscope-software/zen.html#introduction<br>RRID:SCR_013672<br>HelioScan 2-photon imaging software: https://github.com/HelioScan/HelioScan
Data analysis	R Version v3.6.3. The R Project for Statistical Computing http://www.r-project.org/<br>RRID:SCR_001905<br>GraphPad Prism Version v9.1.1. GraphPad http://www.graphpad.com/<br>RRID:SCR_002798<br>iGraph package Version 1.2.6. http://igraph.org/r/<br>RRID:SCR_019225<br>Fiji/ImageJ Version version 2.9.2 Fiji http://fiji.sc<br>RRID:SCR_002285

For manuscripts utilizing custom algorithms or software that are central to the research but not yet described in published literature, software must be made available to editors and reviewers. We strongly encourage code deposition in a community repository (e.g. GitHub). See the Nature Portfolio guidelines for submitting code & software for further information.

Data

Policy information about availability of data

All manuscripts must include a data availability statement. This statement should provide the following information, where applicable:

- Accession codes, unique identifiers, or web links for publicly available datasets
- A description of any restrictions on data availability
- For clinical datasets or third party data, please ensure that the statement adheres to our policy

Data generated and analysed during this study are included in this published article (and its supplementary information and source files) or available from the corresponding author on reasonable request. Data for lineage analysis are available at https://github.com/JessbergerLab/AgingNeurogenesis_Imaging.

Human research participants

Policy information about studies involving human research participants and Sex and Gender in Research.

Reporting on sex and gender	n/a
Population characteristics	n/a
Recruitment	n/a
Ethics oversight	n/a

Note that full information on the approval of the study protocol must also be provided in the manuscript.

Field-specific reporting

Please select the one below that is the best fit for your research. If you are not sure, read the appropriate sections before making your selection.

☒ Life sciences ☐ Behavioural & social sciences ☐ Ecological, evolutionary & environmental sciences

For a reference copy of the document with all sections, see nature.com/documents/nr-reporting-summary-flat.pdf

Life sciences study design

All studies must disclose on these points even when the disclosure is negative.

Sample size	Sample size were derived from previous studies (please refer to methods section) such as Pilz et al., 2018 Science (doi: 10.1126/science.aao5056) and Bottes et al., 2021 Nature Neuroscience (doi: 10.1038/s41593-020-00759-4).
Data exclusions	No data were excluded.
Replication	Experiments were replicated in at least three biological replicates (for details please refer to methods sections). Replication experiments were successful.
Randomization	No randomization for intravital imaging was possible due to comparison of two age groups.
Blinding	Experimenters were blinded for group allocation during analyses (for details please refer to methods sections). Experimenters were not blinded for group allocation during data acquisition for intravital imaging as two age groups were compared (the imaging was performed at different times). Experimenters were blinded for data acquisition of the two age-groups for all analyses on fixed tissues (for details refer to the methods section).

Reporting for specific materials, systems and methods

We require information from authors about some types of materials, experimental systems and methods used in many studies. Here, indicate whether each material, system or method listed is relevant to your study. If you are not sure if a list item applies to your research, read the appropriate section before selecting a response.

Materials & experimental systems

n/a	Involved in the study
☐	☒ Antibodies
☒	☐ Eukaryotic cell lines
☒	☐ Palaeontology and archaeology
☐	☒ Animals and other organisms
☒	☐ Clinical data
☒	☐ Dual use research of concern

Methods

n/a	Involved in the study
☒	☐ ChIP-seq
☒	☐ Flow cytometry
☒	☐ MRI-based neuroimaging

Antibodies

Antibodies used

See Supplementary Table 1:
Primary
Antigen Host Source Catalog #
GFP Goat Rockland 600-101-215
Ki67 Rat Thermo Fisher Scientific 14-5698-82
Dcx Guinea pig Millipore ab2253
GFAP Chicken Aves GFAP
Sox2 Rabbit Millipore AB5603
Sox2 Rat Thermo Fisher Scientific 14-9811-82
S100b Rabbit abcam ab52642
tdTomato Goat Scgen AB8181-200

Secondary
Antigen Host Source Catalog #
Alexa Fluor 488 anti-goat IgG (H+L) Donkey Jackson Immuno Research 705-545-147
Alexa Fluor 488 anti-chicken IgG (H+L) Donkey Jackson Immuno Research 703-545-155
Alexa Fluor 488 anti-rabbit IgG (H+L) Donkey Jackson Immuno Research 711-545-152
Alexa Fluor 488 anti-rat IgG (H+L) Donkey Jackson Immuno Research 712-545-153
Alexa Fluor Cy3 anti-rat IgG (H+L) Donkey Jackson Immuno Research 712-165-153
Alexa Fluor Cy3 anti-goat IgG (H+L) Donkey Jackson Immuno Research 705-165-147
Alexa Fluor 647 anti-rabbit IgG (H+L) Donkey Jackson Immuno Research 711-605-152
Alexa Fluor 647 anti-guinea pig IgG (H+L) Donkey Jackson Immuno Research 706-605-148

Validation

Validation links and references for primary antibodies:
GFP Goat Rockland 600-101-215 https://scicrunch.org/resolver/AB_218182
Ki67 Rat Thermo Fisher Scientific 14-5698-82 https://scicrunch.org/resolver/AB_10854564
Dcx Guinea pig Millipore ab2253 https://www.merckmillipore.com/CH/de/product/Anti-Doublecortin-Antibody,MM_NF-AB2253
GFAP Chicken Aves GFAP https://scicrunch.org/resolver/AB_2313547/mentions?q=&i=rrid:ab_2313547-127:gfap
Sox2 Rabbit Millipore AB5603 https://scicrunch.org/resolver/RRID:AB_2286686
Sox2 Rat Thermo Fisher Scientific 14-9811-82 https://scicrunch.org/resolver/AB_11219471
S100b Rabbit abcam ab52642 https://scicrunch.org/resolver/AB_882426
tdTomato Goat Scgen AB8181-200 https://ecom.masterinsoft.com/catalog/product/AB8181

Animals and other research organisms

Policy information about studies involving animals; ARRIVE guidelines recommended for reporting animal research, and Sex and Gender in Research

Laboratory animals

Ai14;B6.Cg-Gt(ROSA)26Sortm14 (CAG-tdTomato)Hze; The Jackson Laboratory, 007914
(used to breed experimental Gli1-CreERT2::Rosa26-LSL-tdTomato mice)
Gli1tm3(cre/ERT2)Alj; The Jackson Laboratory, 007913
(used to breed experimental Gli1-CreERT2::Rosa26-LSL-tdTomato mice)
Gli1-CreERT2::Rosa26-LSL-tdTomato (e.g., Pilz et al., 2018 Science (doi: 10.1126/science.aao5056)
Experiments started in 2-month-old and 12-14-month-old mice
B6.Cg-Tg(Nes-EGFP)1Yamm/Rbrc
2-month-old and 12-month-old mice were used.
Mice were group housed in ventilated cages (21-23 Celsius, 40-60% humidity) under a 12h dark/light cycle with ad libitum access to food and water.

Wild animals

No wild animals were used in this study.

Reporting on sex

Mice of mixed sex were used.

Field-collected samples

No field-collected samples were used in this study.

Ethics oversight

Animal experiments were approved by the Cantonal Commission for Animal Experimentation of the Canton of Zurich, Switzerland in accordance with national and cantonal regulations (license numbers ZH037/17; ZH190/19; ZH126/20) .

Note that full information on the approval of the study protocol must also be provided in the manuscript.
